# Heteromeric HSFA2/HSFA3 complexes drive transcriptional memory after heat stress in *Arabidopsis*

**DOI:** 10.1038/s41467-021-23786-6

**Published:** 2021-06-08

**Authors:** Thomas Friedrich, Vicky Oberkofler, Inês Trindade, Simone Altmann, Krzysztof Brzezinka, Jörn Lämke, Michal Gorka, Christian Kappel, Ewelina Sokolowska, Aleksandra Skirycz, Alexander Graf, Isabel Bäurle

**Affiliations:** 1 Institute for Biochemistry and Biology, University of Potsdam, Potsdam, Germany; 2 Max-Planck-Institute for Molecular Plant Physiology, Potsdam, Germany; 3 Present Address: School of Life Sciences, University of Dundee, Dundee, UK

**Keywords:** Epigenetics, Plant molecular biology, Plant stress responses, Heat

## Abstract

Adaptive plasticity in stress responses is a key element of plant survival strategies. For instance, moderate heat stress (HS) primes a plant to acquire thermotolerance, which allows subsequent survival of more severe HS conditions. Acquired thermotolerance is actively maintained over several days (HS memory) and involves the sustained induction of memory-related genes. Here we show that *FORGETTER3*/ *HEAT SHOCK TRANSCRIPTION FACTOR A3* (*FGT3*/*HSFA3*) is specifically required for physiological HS memory and maintaining high memory-gene expression during the days following a HS exposure. *HSFA3* mediates HS memory by direct transcriptional activation of memory-related genes after return to normal growth temperatures. HSFA3 binds HSFA2, and in vivo both proteins form heteromeric complexes with additional HSFs. Our results indicate that only complexes containing both HSFA2 and HSFA3 efficiently promote transcriptional memory by positively influencing histone H3 lysine 4 (H3K4) hyper-methylation. In summary, our work defines the major HSF complex controlling transcriptional memory and elucidates the in vivo dynamics of HSF complexes during somatic stress memory.

## Abstract (web summary)

Moderate heat stress primes plants to acquire tolerance to subsequent, more severe heat stress. Here the authors show that the HSFA3 transcription factor forms a heteromeric complex with HSFA2 to sustain activated transcription of genes required for acquired thermotolerance by promoting H3K4 hyper-methylation.

## Introduction

Many organisms are frequently exposed to adverse environmental conditions that interfere with their development and growth and are referred to as stress. Plants can acclimate to stress conditions, and a transient stress cue can prime plants for a modified defense response upon exposure to a recurring stress after a stress-less interval^1–3^. This so-called somatic stress memory has been described in response to a number of different biotic and abiotic stress cues (reviewed in refs. ^3–5^). Occasionally, stress-memory may also extend into future generations^6–8^. Somatic transcriptional memory based on enhanced re-induction of stress-induced genes following a second stress exposure has been reported for drought stress^9,10^, salt stress^11^ and for defense-related priming^12–14^. In these cases chromatin modifications, in particular histone H3 lysine 4 (H3K4) methylation have been correlated with transcriptional memory^9,11–13^. However, the mechanistic basis of stress-induced transcriptional memory and its conservation across different phenomena remains poorly understood.

A major factor limiting global crop yields is heat stress (HS), and it is predicted that its prevalence will increase with climate change^15,16^. In response to acute HS, plants acquire thermotolerance and this is molecularly very similar to the HS response (HSR) of yeast and metazoans^17–20^. However, in nature, HS is often recurring, and plants can be primed by one HS for an improved response to a recurring HS after a stress-less lag phase of several days^3,21,22^. This HS memory is an active process as it is genetically separable from the acquisition of thermotolerance, and several genes have been identified that function specifically in HS memory^21–25^.

An essential component of transcriptional HS responses across kingdoms is their activation through HEAT SHOCK FACTOR (HSF) transcription factors. Interestingly, the activity of HSF proteins is also highly relevant for aging and tumorigenesis^18,19^. While yeast only has one HSF and vertebrates have up to 4, this gene family has radiated massively in higher plants^26^. Of the 21 HSF genes in *Arabidopsis thaliana*, seven have been implicated in the HSR, among them three isoforms of *HSFA1*; *A1A*, *A1B*, and *A1D*^26–31^. The *HSFA1* genes are considered as master regulators that function in a largely analogous manner to yeast and metazoan HSF1^20^. *HSFA1* isoforms are constitutively expressed and are posttranslationally activated upon HS. They induce a suite of target genes, including many heat shock proteins (HSPs) that act as chaperones. HSFA2 specifically functions in HS memory^22^ and it is very strongly induced after HS by HSFA1 proteins^28,29^. HSFA2 in turn amplifies the transcriptional induction of a subset of HS-response genes, but is not required for their initial activation. This subset overlaps with the genes that have been classified as HS memory-related genes due to their sustained induction after HS, lasting for at least two days^23^. Interestingly, HSFA2 binds only transiently to these HS memory-related genes, while HSFA2-dependent differences in transcriptional activity are mostly observed after binding of HSFA2 has decreased^32^. Chromatin profiling of HS memory-related genes revealed that HSFA2 recruits H3K4 hyper-methylation at these loci, and this correlates with the duration of the memory phase (at least 5 d)^32,33^. This HS-induced enrichment likely extends the phase of active transcription at these genes and was not present in highly HS-inducible “non-memory” genes such as *HSP70* and *HSP101*. Thus, the current model is that HSFA2 sustains transcriptional activation through the memory phase by recruiting sustained H3K4 methylation. This mediates a transcriptional memory (type I) that sustains transcriptional activity of certain genes for several days after the end of a short HS. After transcription has subsided, a second type of transcriptional memory remains active at a subset of genes. This causes enhanced transcriptional re-induction upon a recurring HS (type II transcriptional memory) and is active for 6 d after the priming HS^33^. HSFA2 is required for both types of transcriptional memory after HS^32^. However, the mechanistic basis of how HSFA2 promotes HS memory remains poorly understood. It is well established that HSF proteins form trimeric and hexameric complexes in yeast, metazoans and plants^18,34,35^. Yet, major unresolved questions are (1) whether HSFA2 is the only HSF protein in *A. thaliana* that mediates HS memory, and (2) what the composition of the HSFA2-containing HSF complexes is. (3) More generally, the composition of HSF complexes in vivo at endogenous expression levels is virtually unknown, as is the function of many of the different HSF family members in *A. thaliana*.

To identify additional components required for regulation of HS memory, we have employed a reporter-based genetic screen where the HS memory gene *HSA32* was translationally fused to the *LUCIFERASE* reporter gene^24^. *HSA32* shows sustained induction after HS and the corresponding mutant is specifically defective in HS memory at the whole plant level^21,24^. Screening for mutants with normal activation but reduced maintenance of *HSA32-LUC* expression, we identified the *forgetter1* (*fgt1*) mutant^24^. *FGT1* encodes the *A. thaliana* orthologue of Drosophila *strawberry notch*, a highly conserved helicase protein that is required to maintain an open chromatin conformation through cooperation with chromatin remodeling complexes of the SWI/SNF family^24,36^.

Here, we describe the isolation and characterization of the *FGT3* gene from the above screen. We show that *FGT3* encodes the *HSFA3* gene and that the *fgt3* mutant has a HS memory-specific phenotype, comparable to *hsfa2*. We provide evidence that *HSFA3* is a second key HSF underlying HS memory and that it forms heteromeric complexes with HSFA2 that efficiently promote transcriptional memory. These findings serve not only to assign function to a further important HSF family member, but also provide information about the in vivo composition of HSF complexes in *A. thaliana*.

## Results

### *FORGETTER3 (FGT3)* is required for HS memory and sustained induction of *HSA32*

**Fig. 1:**
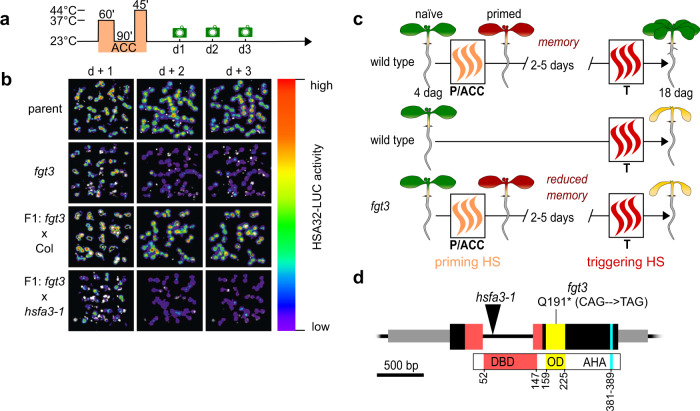
*FGT3*/*HSFA3* is required for HS memory. **a** Treatment scheme for LUC-based HS memory assay: 4 d-old seedlings were exposed to a two-step acclimation treatment (ACC; 60 min 37 °C, 90 min 23 °C and 45 min 44 °C). Activity of the HS memory marker *pHSA32::HSA32-LUC* was scored on the following three days (green camera symbols). **b** LUC-based HS memory assay shows reduced maintenance of *pHSA32::HSA32-LUC* induction in *forgetter 3 (fgt3)* mutants. Crossing to Col but not to the *hsfa3-1* mutant complements the defect of *fgt3* in the F1 progeny. **c** Schematic representation of physiological HS memory: Plants that have not experienced any HS (naïve plants) can be primed by a non-lethal HS (P or ACC), leading to an enhanced capacity to withstand a triggering HS (T). This enhanced thermotolerance results in increased survival of T in a primed plant compared to a naïve plant for up to 5 d (HS memory). *Fgt3* mutants are defective in HS memory and do not survive the T despite prior priming. **d** Schematic representation of the *HSFA3* locus (*At5g03720*) with location of the *fgt3* (Q191*) and *hsfa3-1* mutations. Exons are shown as large black boxes with protein domains overlaid in color (DBD: DNA-binding domain, OD: oligomerization domain, AHA: AHA motif), aa numbers are given to depict the positions of protein domains. UTRs are shown as gray boxes and the intron as a black line.

To identify factors that are implicated in HS memory, we performed a mutagenesis screen for modifiers of HS-induced sustained expression of *pHSA32::HSA32-LUC*^24^. In *fgt3* mutants HSA32-LUC induction was normal at 1 d after a two-step HS treatment (“acclimation”, ACC, Fig. 1a), but declined prematurely during the following two days at normal growth temperature (Fig. 1b). In line with this finding *fgt3* mutants also had a defective HS memory at the physiological level (Fig. 1c, Supplementary Fig. 1a). However, the immediate HS responses - as assayed by basal thermotolerance and acquired thermotolerance - were not affected in *fgt3* (Supplementary Fig. 1b-c). Thus, *FGT3* is specifically required for HS memory.

### *FGT3* encodes *HSFA3*

**Fig. 2:**
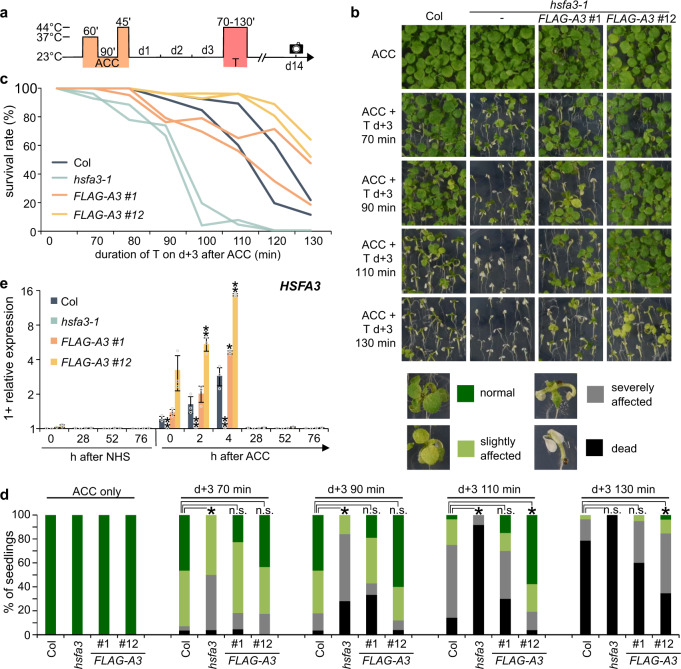
*FGT3*/*HSFA3* is required for physiological HS memory. **a** Treatment scheme for HS memory assays: plants are exposed to a triggering HS (T, 44 °C for 70–130 min) 3 d after ACC (which was applied 4 d after germination) and survival is scored 14 d after ACC (black camera symbol). **b**–**d** HS memory assay of *hsfa3-1* and two complementing lines of *pHSFA3::FLAG-HSFA3* in the *hsfa3-1* background. **b** Representative images of HS memory assay, with legend showing examples of phenotype categories used for the quantification in **d**. **c** Survival rates of the different genotypes in HS memory assay. Data are from 2 independent replicates with *n* ≥ 19 seedlings for each timepoint and genotype. **d** Distribution of phenotypic categories observed in the HS memory assay shown in **b**. Asterisks depict significant differences to Col (*p* < 0.01, Fisher’s exact test, *n* ≥ 19 seedlings for each timepoint and genotype). **e** Transcript levels of *HSFA3* in Col, *hsfa3-1* and two *pHSFA3::FLAG-HSFA3* lines in the *hsfa3-1* background as measured by qRT-PCR. Expression values are relative to the *At4g26410* reference gene, as in all following qRT-PCR figures. Data are mean ± SD of three independent experiments and asterisks indicate significant differences to Col (*, *p* < 0.05; **, *p* < 0.01; unpaired two-sided t-test).

The *fgt3* mutant segregated as a single recessive locus with no apparent morphological defects under normal growth conditions. To identify the genetic mutation underlying the HS memory phenotype, we combined recombination breakpoint mapping and genome re-sequencing, and identified a single nucleotide polymorphism in exon 2 of *At5g03720* that introduces a premature stop codon in the *HSFA3* gene (Fig. 1d). To confirm that *fgt3* is allelic to *hsfa3*, we crossed *fgt3* to *hsfa3-1* and assayed HSA32-LUC activity in the F1 progeny after HS. The progeny of the *fgt3* x *hsfa3-1* cross, but not the control cross, showed strongly reduced maintenance of HSA32-LUC activity and loss of physiological HS memory, similar to *fgt3* (Fig. 1b, Supplementary Fig. 2a). In addition, genomic constructs expressing *HSFA3* under the control of the endogenous promoter (1.3 kb promoter fragment) with or without an N-terminal FLAG tag complemented *HSA32-LUC* expression and survival phenotypes of *fgt3* (Supplementary Figs. 1a, 2b). The *hsfa3-1* allele displayed similar HS memory defects as *fgt3* and was also complemented by *pHSFA3::FLAG-HSFA3* (Fig. 2a–d). Thus, the loss of *HSFA3* function is causative for the *fgt3* mutant phenotypes and we renamed *fgt3* as *hsfa3-3*.

Under our conditions, *HSFA3* induction peaked at 4 h after the end of ACC (Fig. 2e). We also tested the expression pattern of the two *pHSFA3::FLAG-HSFA3* lines. Line #1 expressed *FLAG-HSFA3* similarly as *HSFA3* in Col. Line #12, however, showed a several-fold stronger induction of *FLAG-HSFA3* after ACC, suggesting that this line acts as a native overexpressor (Fig. 2e). Interestingly, line #12 displayed enhanced HS memory compared to Col (Fig. 2b). Further extending the recovery phase between the priming and triggering HS for up to 6 d and reducing the dose (duration) of the triggering HS revealed that HS memory in wild type was still detected after 5 d of recovery, albeit at decreasing levels (Supplementary Fig. 3a, b). This observation extends the memory period regarding physiological effects by 2 d compared to previous reports^23,24^. Notably, the native overexpressing line retained some HS memory 6 d after ACC, when primed Col plants no longer had enhanced survival compared to unprimed Col plants. Thus, HSFA3 protein levels control HS memory.

### *HSFA3* and *HSFA2* interact genetically and have redundant and non-redundant functions

**Fig. 3:**
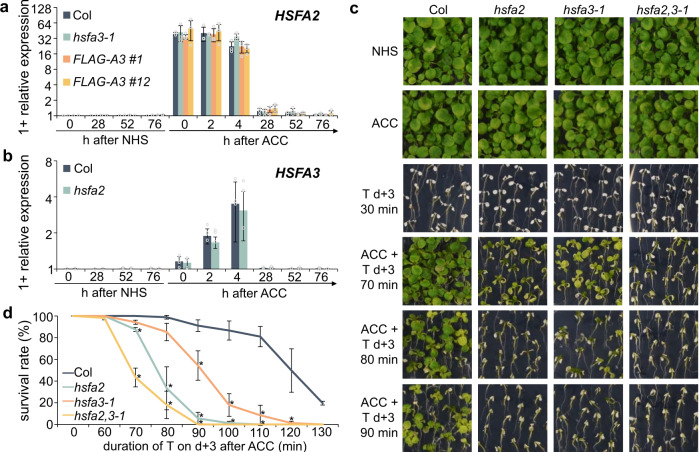
*HSFA2* and *HSFA3* are independently induced by HS and interact genetically. **a** Relative transcript levels of *HSFA2* in Col, *hsfa3-1* and *pHSFA3::FLAG-HSFA3* lines in *hsfa3-1* background as measured by qRT-PCR. **b** Relative transcript levels of *HSFA3* in Col and *hsfa2* as measured by qRT-PCR. Data are mean ± SD of three independent experiments (**a**, **b**). **c**, **d** HS memory assay for *hsfa2*, *hsfa3-1* and *hsfa2 hsfa3-1* double mutants. 4 d-old seedlings were exposed to ACC treatment and 3 d later to a triggering HS at 44 °C for 70–130 min. NHS, no-HS control; ACC, plants primed with an ACC treatment. Representative images (**c**) and survival rates (**d**) were recorded 14 d after ACC. Error bars indicate SD of three independent experiments. Asterisks mark significant differences to Col (*p* < 0.05, unpaired two-sided t-test).

*HSFA3* was induced by ACC, albeit more slowly than *HSFA2*, which peaked right at the end of the ACC treatment (Fig. 3a, b). *HSFA3* was suggested to be induced by the HS-activated DEHYDRATION-RESPONSIVE ELEMENT BINDING PROTEIN 2 A (DREB2A), which in turn is activated by HSFA1 isoforms^37–39^. Indeed, under our HS regime *HSFA3* but not *HSFA2* expression depended on DREB2A, consistent with the predicted presence of more DREB binding elements in the *HSFA3* promoter than in the *HSFA2* promoter (Supplementary Fig. 3c, Supplementary Table 1). This two-step activation may account for the slower induction kinetics of *HSFA3*. Consistent with both genes being downstream of HSFA1s, induction of either *HSFA2* or *HSFA3* was independent of the respective other protein (Fig. 3a, b).

*HSFA2* and *HSFA3* are both required for HS memory, with *hsfa2* having a slightly stronger defect (Fig. 3c, d). To test whether both genes interact genetically, we generated the *hsfa2 hsfa3-1* double mutant and analyzed physiological HS memory. The double mutant was more sensitive to a triggering HS that was applied 3 d after ACC than either single mutant (Fig. 3c, d). None of the mutants or native overexpressing lines showed any defects in basal or acquired thermotolerance, indicating that the observed phenotypes are specific to HS memory (Supplementary Fig. 4). In summary, despite the already strong phenotypes of the single mutants, double mutant analysis indicates that HSFA2 and HSFA3 act partially redundantly.

### *HSFA3* is required for sustained induction of HS memory-related genes

**Fig. 4:**
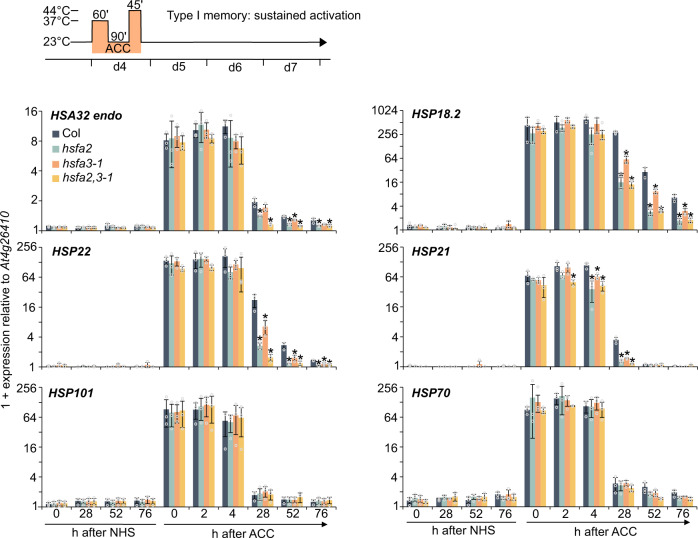
Sustained induction of HS memory genes depends on *HSFA2* and *HSFA3*. Type I transcriptional memory (sustained induction): Memory gene expression is induced by a priming ACC treatment and expression is sustained above baseline for several days. Plants were exposed to an ACC treatment and samples taken at the indicated time points during the following 76 h. Relative transcript levels of 4 memory genes (*HSA32*, *HSP18.2*, *HSP22*, *HSP21*) and 2 HS-inducible non-memory genes (*HSP101* and *HSP70*) were measured by qRT-PCR. Time points depict hours after end of ACC. Data are mean ± SD of three independent experiments. Asterisks mark significant differences to Col (*p* < 0.01, unpaired two-sided t-test).

Two types of HS-related transcriptional memory have been described; type I memory (sustained induction) and type II memory (enhanced re-induction)^5,32^. We asked whether *HSFA3* is required for type I memory (sustained induction) by analyzing transcript levels of the endogenous *HSA32* gene as well as three other genes of this group, *HSP18*.2, *HSP22*, and *HSP21*^23,24,32^. Starting from 28 h after ACC, transcript levels of these genes in *hsfa3-1* were lower compared to wild type, indicating a defect in sustained induction, but not initial upregulation (Fig. 4). In contrast, the native overexpressing line #12 showed increased expression levels of *HSA32*, *HSP22*, and *APX2* from 28 h onwards (Supplementary Fig. 5a), in line with the stronger *HSFA3* expression after ACC and the enhanced HS memory (Fig. 2). In the *hsfa2 hsfa3-1* double mutant, *HSA32*, *HSP22*, and *HSP21* had further reduced transcript levels starting from 28 h compared to either single mutant (Fig. 4). This is in line with the further reduced HS memory in the double mutant and suggests that both proteins act in HS memory and cannot replace each other. In addition, we observed similar changes at the level of unspliced transcripts (Supplementary Fig. 5b), which are often used as a proxy for transcriptional activity^23,24,40^, indicating that the observed changes in transcript levels reflect changes in (ongoing) gene transcription. The expression of HS-induced non-memory genes *HSP101* and *HSP70* was unaltered in all of the mutants (Fig. 4).

**Fig. 5:**
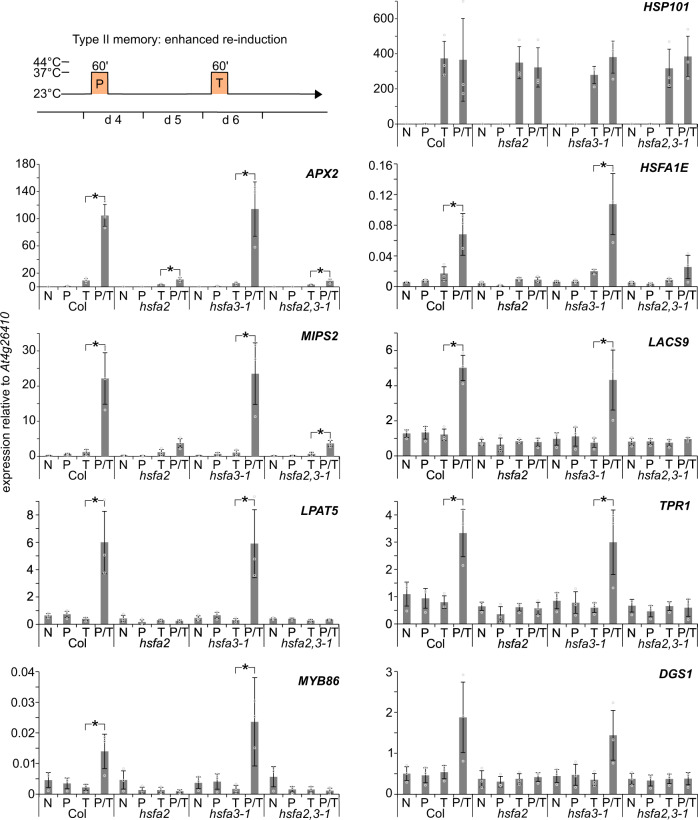
Enhanced re-induction of HS memory genes depends on *HSFA2* but not *HSFA3*. Type II transcriptional memory (enhanced re-induction): Memory gene expression is activated by a priming treatment (P), and more highly activated by a second triggering treatment (T) 2 d later. P and T treatments consist of 37 °C for 1 h. Relative transcript levels in enhanced re-induction experiments of eight memory genes (*APX2*, *HSFA1E*, *MIPS2*, *LACS9*, *LPAT5*, *TPR1*, *MYB86,* and *DGS1*) and one non-memory gene (*HSP101*) were measured by qRT-PCR. Plants were either not treated (N), only primed on d 4 (P), only triggered on d 6 (T), or primed on d 4 and triggered on d 6 (P + T). Regardless of their treatment, all samples were harvested on d 6 at the end of the T treatment. Data are mean ± SD of three independent experiments. Asterisks mark significant differences to Col (*p* < 0.01, unpaired two-sided t-test).

Consistent with previous findings^33^, *HSFA2* was required for type II memory at *APX2, HSFA1E, MIPS2, LACS9*, *LPAT5, TPR1*, *MYB86,* and *DGS1* (Fig. 5). In contrast, *hsfa3-1* mutants showed wild type-like enhanced re-induction of these genes. Expression of *HSP101*, which does not show type II transcriptional memory, was unaffected in all mutant backgrounds (Fig. 5). Thus, while *HSFA2* is required for both types of transcriptional memory, *HSFA3* appears specifically required for the sustained induction of HS memory-related genes (type I).

**Fig. 6:**
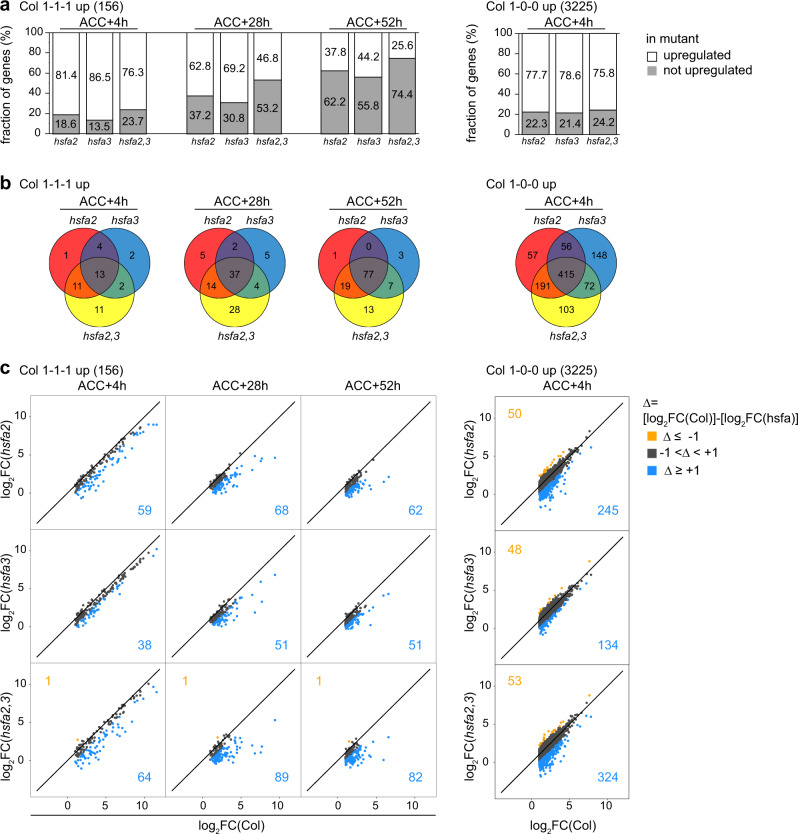
HSFA2 and HSFA3 jointly promote type I transcriptional memory in a genome-wide manner. Genome-wide transcriptome profiling by RNA-seq analysis identifies 156 HS memory genes with sustained induction above no-HS levels (log_2_FC > 1, p < 0.05) at 4 h (ACC + 4 h), 28 h (ACC + 28 h), and 52 h (ACC + 52 h) after end of ACC treatment in Col (1-1-1 up) and 3225 genes that are induced above no-HS levels only 4 h after end of ACC treatment in Col (1-0-0 up). **a** Fraction of 1-1-1 up (Col) and of 1-0-0 up (Col) genes that are no longer upregulated in *hsfa2*, *hsfa3-1* (hsfa3), and *hsfa2 hsfa3-1* (hsfa23) mutants relative to their no-HS expression (log_2_FC ≤ 1 OR log_2_FC > 1 AND *p* > 0.05, blue; log_2_FC > 1 AND *p* > 0.05, gray). **b** Overlap of genes with loss of upregulation relative to no-HS expression (log_2_FC ≤ 1 OR log_2_FC > 1 AND p > 0.05) in *hsfa2*, *hsfa3-1* (hsfa3), or *hsfa2 hsfa3-1* (hsfa23) mutants among 1-1-1 up (Col) and 1-0-0 up (Col) genes. **c** Pairwise comparison of log_2_FCs relative to no-HS expression between Col and *hsfa2*, *hsfa3-1* (hsfa3) or *hsfa2 hsfa3-1* (hsfa23) mutants of 1-1-1 up (Col) and 1-0-0 up (Col) genes. Genes in orange are more strongly induced in the mutant, genes in blue are less induced in the mutant. Colored numbers indicate the number of genes in the respective group.

To globally assess the effects of the mutants on heat-induced gene expression during the memory phase, we performed RNA-seq on Col, *hsfa2*, *hsfa3*, and double mutant seedlings that were subjected to an ACC treatment and recovered for 4 h, 28 h, or 52 h or control samples without ACC. In Col, we identified 156 genes that showed differential gene induction (log_2_ FC > 1 and *p* < 0.05) at all three timepoints (“1-1-1 up”), and that we therefore call HS memory genes (Supplementary Data 1). In contrast, 3225 genes were induced specifically at 4 h after ACC (“1-0-0 up”). Among the memory genes, 18.6%/13.5%/23.7% were not induced in *hsfa2*/ *hsfa3*/ *hsfa2 hsfa3* at 4 h after ACC (Fig. 6a, Supplementary Data 1). These numbers progressively increased to 37.2%/30.8%/53.2% at 28 h and 62.2%/55.8%/74.4% at 52 h after ACC. Thus, HSFA2 and HSFA3 are partially redundantly required for sustained induction of HS memory genes and their effect becomes more pronounced as the recovery phase progresses. In contrast, of the 3225 early HS genes (1-0-0 up), 22.3%/21.4%/24.2% were not induced at 4 h in *hsfa2*/ *hsfa3*/ *hsfa2 hsfa3*. For both groups of genes and at all three timepoints there was a large overlap between the genes with loss of upregulation in the mutants, confirming that their functions are largely overlapping (Fig. 6b). For the memory genes only, the number of genes with loss of overexpression was increased in the double mutant, confirming the cooperative effect of HSFA2 and HSFA3. The overall similar but stronger effect of the double mutant was also apparent from looking at the effect size of individual genes (Fig. 6c). Among the HS memory genes, all differentially expressed genes showed reduced induction, with the exception of two genes. In contrast, among the early HS genes, enhanced induction was more prevalent (Fig. 6c). In summary, global analysis confirmed that HSFA2 and HSFA3 function as transcriptional activators on an overlapping set of HS memory genes, where they are required for sustained induction of gene expression (type I memory).

### HSFA3 and HSFA2 proteins interact

**Fig. 7:**
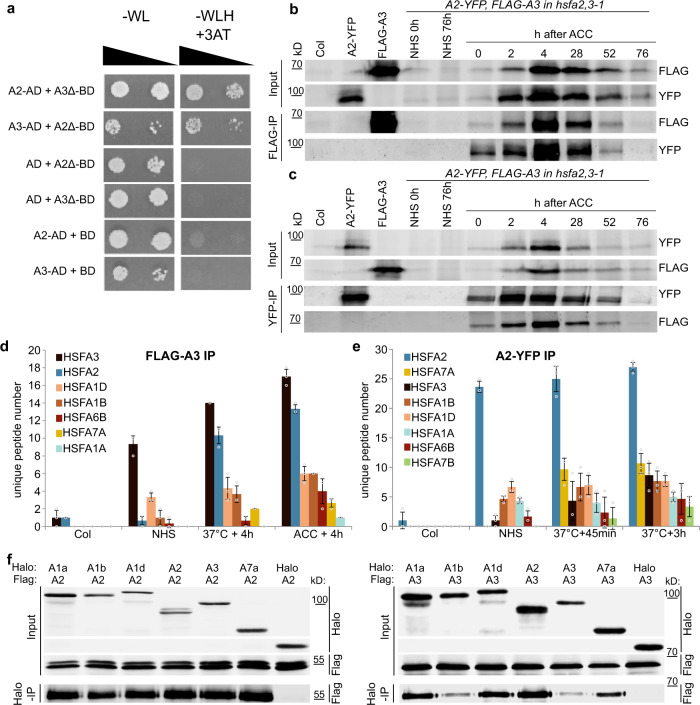
HSFA2 and HSFA3 form protein complexes during HS memory. **a** HSFA2 and HSFA3 interact in the yeast-two-hybrid assay. The C-terminal trans-activating domains of HSFA2 and HSFA3 were deleted (A2**Δ** aa 1-269, A3Δ aa 1-275) when fused to the GAL4-DNA-binding domain (BD) to prevent auto activation. Serial dilutions were grown on –WL medium (not selecting for interaction) or on –WLH medium supplemented with 50 mM 3-AT to check for protein-protein interactions. **b**, **c** HSFA2 and HSFA3 interact *in planta* as shown by co-immunoprecipitation experiments: transgenic lines carrying both *pHSFA2::HSFA2-YFP* and *pHSFA3::FLAG-HSFA3* constructs in the *hsfa2 hsfa3-1* double mutant background were subjected to an ACC treatment and samples were taken at the indicated time points. Immunoprecipitation of FLAG-HSFA3 yielded the HSFA2-YFP protein (**b**) and immunoprecipitation of HSFA2-YFP yielded the FLAG-HSFA3 protein (**c**) at all time points examined. No bands of similar size were co-purified in non-treated plants (NHS 0 h and NHS 76 h), single transgenic lines or Col plants sampled at 4 h after ACC. A representative experiment from 3 independent experiments is shown. **d**, **e** Interacting HSF proteins as identified by co-immunoprecipitation of FLAG-HSFA3 (**d**) or HSFA2-YFP (**e**) followed by mass spectrometry after the indicated treatments (37 °C treatment was for 1 h). Average numbers of unique peptides are given for all HSF proteins identified (cf. Supplementary Table 2). Data are mean ± SD of three independent experiments. Note that the HSF proteins are sorted according to the number of peptides recovered and this differs for HSFA2- and HSFA3-co-purified proteins, the same color code is used in (**d**) and (**e**). **f** In vitro pulldown of HSFA proteins. Pairs of the indicated Halo-tagged and FLAG-tagged HSF proteins were co-translated in vitro and purified with anti-Halo beads. The Halo-tag alone was used as a negative control. A representative experiment from 3 independent experiments is shown.

HSF proteins form multimeric complexes^34,41^. With the plethora of HSF proteins in *A. thaliana*, there is the potential for multiple combinations; however, which of these occur in vivo remains unresolved. We hypothesized that HSFA3 and HSFA2 may directly interact. We confirmed the interaction of HSFA2 and HSFA3 in the yeast-two-hybrid system (Fig. 7a). The C-terminus of HSFA2 and HSFA3, which we truncated in the BD constructs to prevent auto activation, was dispensable for the interaction. This is consistent with the notion that the interaction is mediated by the oligomerization domain (OD, Fig. 1d). We next confirmed the interaction by *in planta* co-immunoprecipitation from stable transgenic lines using HSFA2-YFP^32^ and FLAG-HSFA3, both expressed from their own promoters in the *hsfa2 hsfa3-1* double mutant background. Both proteins were strongly induced after ACC with a peak around 4 h into the recovery phase and they were still detectable at 76 h after ACC (Fig. 7b, c). FLAG-HSFA3 precipitated HSFA2-YFP at all time points where both proteins were detectable (Fig. 7b). Conversely, HSFA2-YFP was able to pull down HSFA3 (Fig. 7c). In summary, HSFA2 and HSFA3 form heteromers *in planta* that persist for several days after HS/ACC.

### Interaction with other HSFs

Our genetic analysis indicated that in the absence of the respective other “memory” HSF, the remaining HSF still retains some activity. Thus, both HSFA2 and HSFA3 may have other binding partners. To investigate this we purified FLAG-HSFA3 and interacting proteins under no-HS conditions, 4 h after HS (1 h at 37 °C), or 4 h after ACC from the complementing *pHSFA3::FLAG-HSFA3* line and subjected them to mass spectrometry analysis (Co-IP/MS). The most frequent interacting protein as estimated by unique peptide numbers in all heat-treated samples was HSFA2 (Fig. 7d, Supplementary Table 2), indicating that HSFA2 is the preferred binding partner of HSFA3. In addition, we also identified HSFA1D, HSFA1B, HSFA6B, HSFA7A, and HSFA1A as interacting proteins (Fig. 7d, Supplementary Table 2). Thus, HSFA3 forms multimeric complexes with other HSF proteins, providing a tentative explanation for the residual activity of HSFA3 in the absence of HSFA2. Interestingly, we also detected interactions of HSFA3 with HSFA1 isoforms before applying any heat treatment, suggesting that the formation of HSFA1-HSFA3 complexes does not depend on a HS stimulus.

Correspondingly, we isolated HSFA2-YFP protein complexes for mass spectrometry (Fig. 7e). Since *HSFA2* expression is induced faster than *HSFA3* (Fig. 3a, b), samples were taken 45 min and 3 h after 1 h HS at 37 °C treatments. HSFA3 was detected with very low peptide numbers under no-HS conditions but increased at 45 min and 3 h after HS in line with the induction of *HSFA3*; at 3 h after HS HSFA3 was the second most frequent interacting protein after HSFA7A. At all time points other HSFs were recovered as HSFA2 interacting proteins. They were HSFA7A, HSFA1B, HSFA1D, HSFA1A, HSFA6B, and HSFA7B (Fig. 7e, Supplementary Table 2). With the exception of HSFA7B, we identified all HSFA2-interacting HSFs also as interactors of HSFA3, suggesting that both proteins share a common set of interactors after HS. Using in vitro pulldowns we confirmed that each HSFA2 and HSFA3 interact directly with HSFA1A, B, D, and HSFA7A (Fig. 7f).

We next asked which proteins (if any) HSFA2 and HSFA3 bind to in the absence of the respective other memory HSF. To this end, we repeated the Co-IP/MS analysis of HSFA2 or HSFA3 in the respective other mutant background in the absence of HS or 4 h after ACC treatment. Besides the memory HSFs, the previously identified additional interacting HSFA1s and HSFA7B were rediscovered (Supplementary Table 3). In the *hsfa2* mutant HSFA3 complexes also contained HSFA1D, HSFA1B and HSFA1A. Conversely, in the *hsfa3* mutant HSFA2 complexes contained HSFA1D, HSFA1B, HSFA1A and HSFA7B. This is consistent with the idea that either memory HSF still forms complexes with additional HSF proteins in the absence of the other memory protein. However, our mutant analysis clearly indicates that these alternative complexes are less efficient in promoting HS memory.

### HSFA3 and HSFA2 bind with overlapping kinetics to the same target genes

**Fig. 8:**
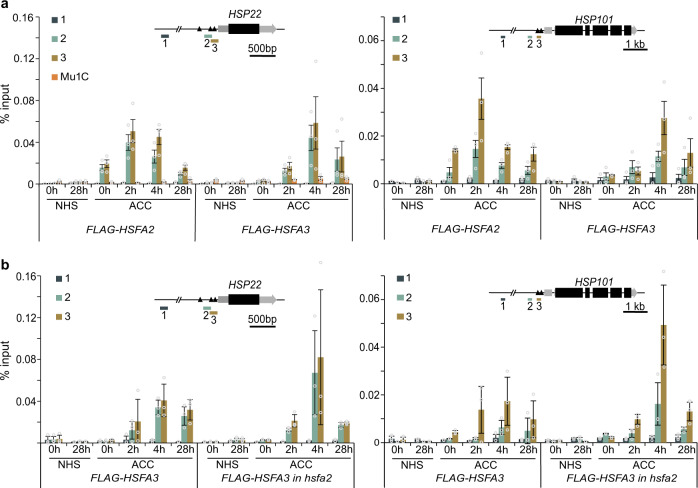
HSFA2 and HSFA3 bind to memory gene promoters directly and independently. **a** Occupancy of HSFA2 and HSFA3 as determined by ChIP-qPCR from *pHSFA2::FLAG-HSFA2* and *pHSFA3::FLAG-HSFA3*. **b** Occupancy of HSFA3 as determined by ChIP-qPCR from *pHSFA3::FLAG-HSFA3* in the wild type or *hsfa2* mutant background. Enrichment normalized to Input (Data are mean ± SD) from three independent experiments is shown for the HS memory gene *HSP22* and the non-memory gene *HSP101*. The transposon *Mu1c* is shown as a non HS-responsive locus. Time points are given in h after end of ACC treatment. For each locus one control amplicon situated approximately 3 kb upstream is shown alongside the amplicon covering heat shock elements (black triangles) in the promoter (inset gene models).

To test whether HSFA3 sustains the expression of HS memory-related genes directly, we performed time-course chromatin immunoprecipitation (ChIP) with sampling from the end of ACC until 28 h into the recovery phase. Indeed, we detected HS-dependent enrichment of FLAG-HSFA3 in the promoters of *HSP22*, *HSP18.2*, *HSA32*, and *APX2* at HSE-containing sequences (Fig. 8a, Supplementary Fig. 6a). The binding of HSFA3 peaked 4 h into the recovery phase, and was still detected at 28 h. This is consistent with HSFA3 promoting transcription for at least 24 h after the end of ACC. We previously found that HSFA2 is associated with these loci early after the HS^32^. We confirmed this in the present study using a *pHSFA2::FLAG-HSFA2* line that was grown and sampled side-by-side with the *FLAG-HSFA3* line (Fig. 8a, Supplementary Fig. 6a). After ACC HSFA3 and HSFA2 were also associated with the HS-inducible non-memory gene *HSP101* (Fig. 8a), where they did not have any impact on gene expression (Figs. 4 and 5). Thus, while HSFA3 and HSFA2 bind to the same loci after HS, their binding kinetics differ with HSFA3 showing a delayed peak. This suggests that both proteins bind these loci with overlapping kinetics, partially together and partially using alternative HSFs as binding partners.

### HSFA3 binds target loci independently of HSFA2

Given the interaction between HSFA2 and HSFA3 we wondered whether binding of HSFA3 to the promoters of target genes depends on HSFA2. We thus performed time-course ChIP with FLAG-HSFA3 in the *hsfa2* background. Overall, the HSFA3 binding dynamics to *HSP22*, *HSP18.2*, *HSA32*, *APX2,* and *HSP101* were very similar to those in the wild type background (Fig. 8b, Supplementary Fig. 6b). This suggests that HSFA2 is not required to recruit HSFA3 to its target loci. Moreover, these findings indicate that the loss of HS memory in *hsfa2* is not due to concomitant loss of HSFA3 for transcriptional activation; rather, it reinforces the idea that alternative HSF complexes have differential capacity to activate HS memory.

### HSFA3 and HSFA2 jointly recruit histone H3K4 methylation

**Fig. 9:**
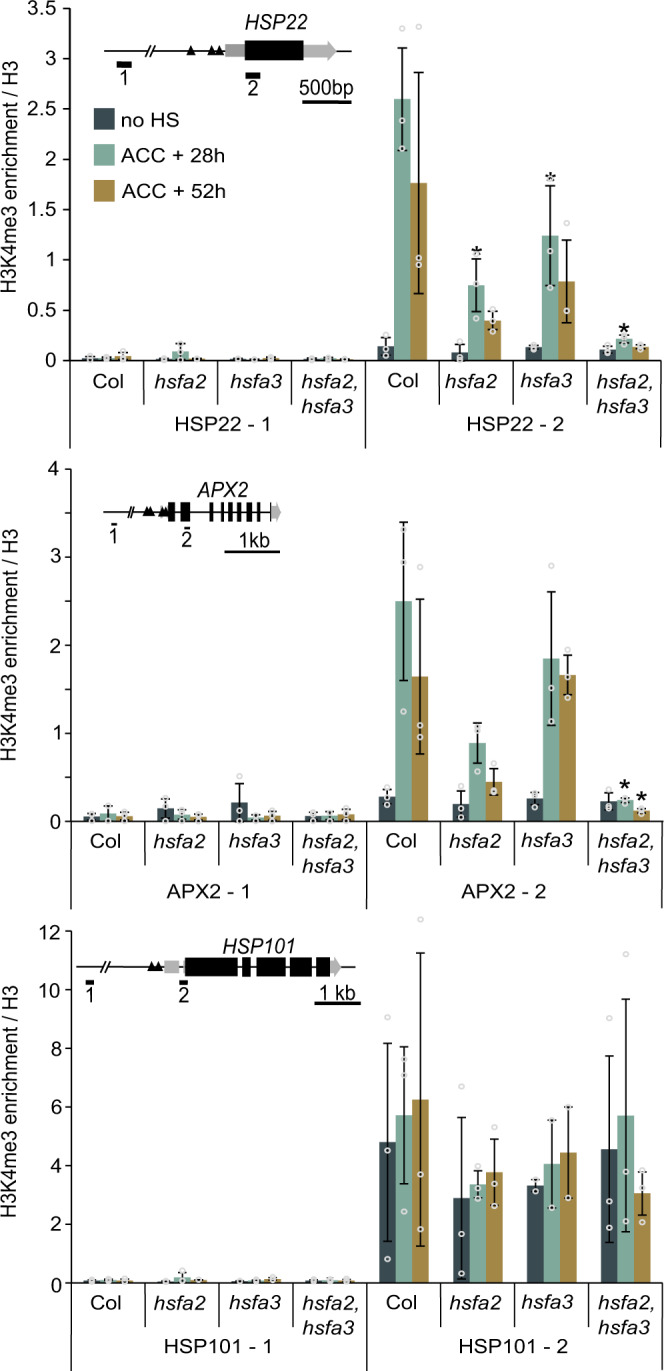
H3K4me3 deposition depends on HSFA2 and HSFA3. Enrichment of H3K4me3 over H3 for the HS memory genes *HSP22* and *APX2*, and the non-memory gene *HSP101* as determined by ChIP-qPCR from three independent experiments. For each locus one control amplicon situated approximately 3 kb upstream is shown alongside the amplicon covering the transcriptional start site (inset gene models). Time points are given in h after end of ACC treatment. Data are mean ± SD. Asterisks mark significant differences relative to Col (*p* < 0.05, unpaired two-sided t-test).

We previously found that *HSFA2* is required for sustained enrichment of H3K4 trimethylation (H3K4me3) at memory-related genes after HS^32^. In *hsfa2* mutants, H3K4me3 enrichment was strongly reduced but not completely abolished. To test the role of HSFA3 in sustained H3K4me3 enrichment, we analyzed H3K4me3 levels in the double mutant and either single mutant at 28 h and 52 h after ACC. Indeed, in either single mutant H3K4me3 enrichment after ACC was reduced to an intermediate level at *HSP22*; at *APX2*, HSFA3 was dispensable for H3K4me3 enrichment, however, at both loci H3K4me3 was more strongly reduced in the *hsfa2 hsfa3-1* double mutant (Fig. 9). In contrast, H3K4me3 enrichment at *HSP101* was not affected in either of the genotypes tested. Over all genotypes and assayed regions, histone H3 enrichment decreased after ACC (Supplementary Fig. 7), consistent with previous findings^24^. In summary, our findings suggest that HSFA2 and HSFA3, despite the strong phenotypes of the single mutants, show functional redundancy at the level of physiological HS memory, sustained memory-related gene activation, and hyper-methylation of H3K4me3.

### HSFA2 and HSFA3 can substitute for each other

**Fig. 10:**
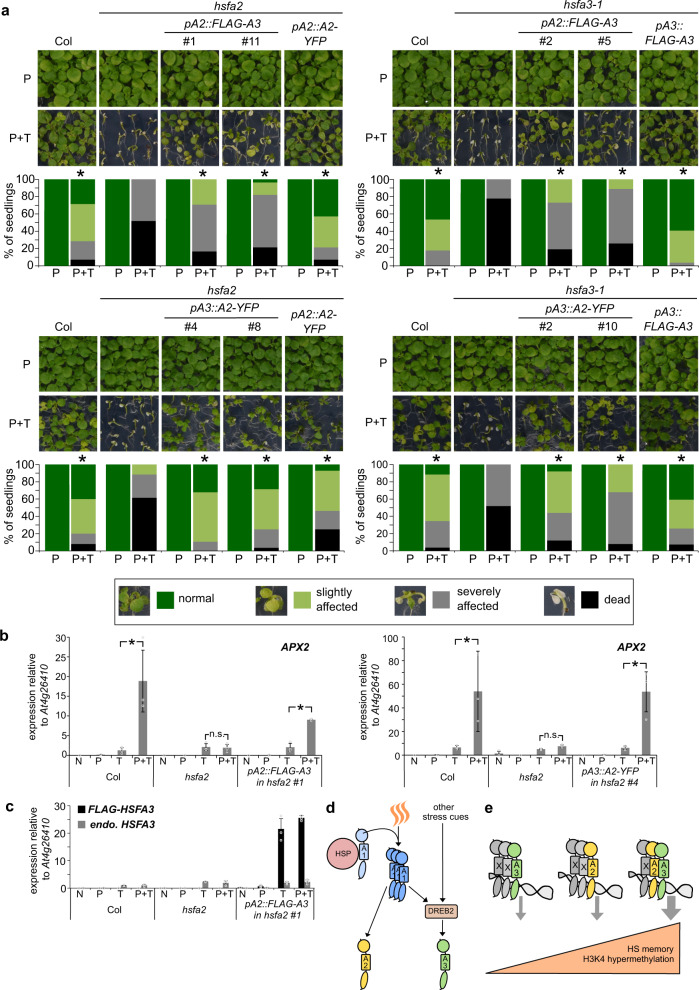
Promoter swapping indicates that HSFA2 and HSFA3 proteins carry out the same functions and working model. **a** *FLAG-HSFA3* expressed from the *HSFA2* promoter complements *hsfa2* and *hsfa3-1* mutants in HS memory assays. *HSFA2-YFP* expressed from the *HSFA3* promoter complements *hsfa2* and *hsfa3-1* mutants in HS memory assays. Asterisks mark significant differences to the mutant background (p < 0.05, Fisher’s exact test, *n* ≥ 24 seedlings for each timepoint and genotype). Phenotype categories used for quantification are indicated. **b**, **c** *pHSFA2::FLAG-HSFA3* and *pHSFA3::HSFA2-YFP* are each sufficient to rescue the type II memory defect of *hsfa2*. Relative transcript levels in enhanced re-induction experiments shown for the memory gene *APX2* (**b**) and endogenous HSFA3 or FLAG-HSFA3 (**c**) as measured by qRT-PCR. Samples were either not treated at all (N), only primed on d 4 (P), only triggered on d 7 (T) or primed on d 4 and triggered on d 7 (P + T). All samples were harvested at the end of the triggering HS (T) on d 7. Data are mean ± SD of three independent experiments. Asterisks mark significant differences for the indicated comparisons (p < 0.01, unpaired two-sided t-test). **d**, **e** Working model for HS memory regulation by HSFA2 and HSFA3. **d** Differential regulation of *HSFA2* and *HSFA3* fine-tunes HS responses and integrates different environmental cues. A priming HS activates HSFA1 proteins through the release from HSPs and formation of active trimers. Active HSFA1s promote the expression of *HSFA2* and *DREB2* genes. DREB2 in turn promotes the expression of *HSFA3*; since DREB2s are also induced and activated at the posttranslational level by other stress cues, this may serve to integrate different cues into the HS memory response through HSFA3. **e** Different HSF complexes containing HSFA2 and/or HSFA3 form after a priming HS. They vary in their capacity to activate HS memory. The most efficient HSF complex to promote HS memory contains both HSFA2 and HSFA3, while complexes with only one of the two proteins have a reduced capacity for the recruitment of H3K4 hyper-methylation and for HS memory.

The strong phenotypes of the single mutants may be caused by partially non-overlapping expression domains or by sub-functionalization at the protein level. To discriminate between these possibilities, we performed complementation analyses with HSFA2 and HSFA3 proteins that were expressed under the control of the respective other promoter. We first expressed the *FLAG-HSFA3* coding region from the *pHSFA2* promoter, which is activated earlier after HS than *pHSFA3* (cf. Fig. 3a, b). This construct was able to partially complement the physiological HS memory phenotype of *hsfa2* (Fig. 10a, Supplementary Fig. 8a, d), suggesting that the HSFA3 protein can (partially) take over HSFA2 function, when supplied from the *HSFA2* promoter. The *pHSFA2::FLAG-HSFA3* construct also partially complemented the *hsfa3-1* mutant (Fig. 10a). We conversely asked whether the early induction of *HSFA2* is required for HS memory by expressing the complementing *HSFA2-YFP* coding region under the control of *pHSFA3*. Indeed, *pHSFA3*- and *pHSFA2*-driven *HSFA2-YFP*, respectively, rescued the *hsfa2* mutant phenotype in part (Fig. 10a). Finally, expression of *HSFA2-YFP* from *pHSFA3* was sufficient to restore HS-memory in *hsfa3-1*, suggesting that both proteins carry out the same function. In contrast, expressing *FLAG-HSFA1D* from the *pHSFA3* promoter failed to complement the *hsfa3-1* mutant (Supplementary Fig. 8e–g), indicating that HSFA2 and HSFA3 have a specialized protein function that is absent from HSFA1D.

*HSFA2* but not *HSFA3* is required for type II transcriptional memory after HS (Fig. 5). This is surprising in light of the above finding that both proteins appear to carry out the same functions. To further investigate this, we tested whether HSFA3 could substitute for HSFA2 regarding type II memory if expressed from *pHSFA2*. Introduction of *pHSFA2::FLAG-HSFA3* into *hsfa2* restored the enhanced re-induction of *APX2* after recurring HS, suggesting that *HSFA3* is able to mediate type II memory when supplied under the correct promoter (Fig. 10b, c). Conversely, *pHSFA3::HSFA2-YFP* rescued type II transcriptional memory defects of *APX2* in the *hsfa2* mutant. In summary, the promoter swapping experiments indicate that there is no clear qualitative difference between HSFA2 and HSFA3 protein functions. In the absence of HSFA2-HSFA3 heteromers, increased protein levels and correct timing of expression partially compensate for the missing partner.

## Discussion

Here, we identified *HSFA3* as an essential component of HS memory in *A. thaliana*. We show that HSFA3 is required for sustained induction of several HS memory-related genes through direct gene activation and recruitment of histone H3K4 hyper-methylation. Previously, only HSFA2 was implicated in HS memory. We demonstrate that HSFA3 binds to HSFA2 to form heteromeric complexes that are highly effective at promoting HS memory.

*HSFA2* and *HSFA3* show different expression dynamics and this may allow fine-tuning of HS (memory) responses according to the actual environmental conditions. HSFA2 is a direct target gene of HSFA1 and induced very rapidly after the onset of HS^22,30^. In contrast, HSFA1s or HSFA2 do not directly induce HSFA3 (Fig. 10d). Rather, HSFA3 is activated by DREB2A, which in turn is activated by HSFA1A, and the related DREB2B and DREB2C^30,37–39^. DREB2A and DREB2B are also induced by drought and high salinity stress, and DREB2A is in addition regulated at the posttranslational level^39,42,43^. Under our conditions, *HSFA3* expression is primarily induced during the recovery phase, which is in full agreement with its function during HS memory. HSFA3 and its presumed activator DREB2B were reported as the only two transcription factors specifically enriched in acclimated plants, but not by direct exposure to acute HS^37,44^. Another study reported *HSFA3* expression peaking after 10 h of continuous 37 °C treatment^38^. These studies provided inconclusive evidence regarding the functional involvement of HSFA3 in HS responses. Our finding that HSFA3 has a specific role in HS memory unifies these studies and assigns a clear function to HSFA3.

To further assess the significance of the different expression dynamics, we performed promoter swapping experiments. Both *HSFA2* and *HSFA3* rescued the other respective mutant when expressed from the corresponding promoter at least partially. While *HSFA3* was not required for enhanced re-induction after a second HS (type II memory), it was able to partially complement the type II memory defects of *hsfa2* when expressed from *pHSFA2*, suggesting that the early induction of *HSFA2* contributes to type II memory. Importantly, HSFA1D was not able to rescue the *hsfa3* mutant, indicating functional specialization at the protein level. Thus, HSFA2 and HSFA3 appear to have similar protein properties enabling them to recruit specific transcriptional co-activators or H3K4 methyltransferases, and these appear distinct from the remainder of the HSF family. It remains an open question whether this recruitment occurs through direct protein-protein interactions or through other proteins, e. g. components of the general transcriptional machinery. H3K4 methylation is deposited by the COMPASS complex and is required for efficient transcriptional elongation^45–48^. This is critical for transcriptional regulation in development and stress response in animals, where release of paused RNA Pol II into elongation is a limiting step^49,50^. Understanding the molecular basis for the memory-specific function of HSFA2 and HSFA3 will be an important goal of future work.

In yeast and animals, HSFs are present as trimers or hexamers (where two trimers combine), and a similar structure has been proposed for plant HSF complexes^18,20,34,35,51^. HSFA2 and HSFA3 associate with each other during the three days following a priming HS. HSFA3 binding to its target sites was independent of HSFA2, suggesting that it also forms functional complexes with other HSFs that may also be represented in the trimeric HSFA2/HSFA3/X complexes. We identified several HSFA1s (1 A, 1B, 1D) as well as HSFA7A, and HSFA6B as direct interactors of both HSFA2 and HSFA3. Indeed, we showed that binding partners of both memory HSFs in the absence of the respective other memory HSF contained the same HSFs that were found in the presence of the other memory HSF. This supports the notion that despite their overall promiscuity, only complexes that contain both HSFA2 and HSFA3 have full capacity to activate HS memory (Fig. 10e). If HSFs assemble in heteromeric trimers with varying components, these complexes may vary in their temporal regulation, co-activator activity and target specificity; they may serve to integrate responses to different environmental cues. Noteworthy, *HSFA3* responds to oxidative stress and both *HSFA2* and *HSFA3* are activated by excess light, while *HSFA6B* is activated by salt stress, osmotic stress and ABA^52–54^.

This work has started to unravel the in vivo complexity and dynamics of plant HSF complexes. HSFA2 and HSFA3 share a specific ability to recruit transcriptional co-activators and histone H3K4 methyltransferases during HS memory, which other HSFA proteins cannot do in their absence. HSFA2 and HSFA3 are found in heteromeric complexes together with additional HSFs, in particular HSFA1s. Maximal HS memory activation likely depends on the formation of heteromeric HSFA2/HSFA3/X trimers (Fig. 10e). The surprisingly strong single mutant phenotypes of *hsfa2* and *hsfa3* support this model; in the mutant backgrounds, trimers only contain a single memory HSF, resulting in much less efficient activation of transcriptional memory (Fig. 10e). The partial complementation of *hsfa2* single mutants by an additional copy of *HSFA3* and vice versa would then be due to a higher overall abundance of such less efficient, single-memory HSF-containing complexes. In summary, our work has begun to shed light on the composition and specialized functions of in vivo HSF complexes in *A. thaliana*, resulting in testable predictions about a super-memory HSF complex. Ultimately, understanding the function of HSF complexes in heat shock response and transcriptional memory at a detailed biochemical level will provide targets for engineering crop plants that are more resilient to temperature extremes.

## Methods

### Plant material and growth conditions

All *A. thaliana* lines used in this study are in the Col-0 background. The *pHSA32::HSA32-LUC*^24^ and *pHSFA2::HSFA2-YFP* lines^32^, the *hsfa2-1*^22^, *hsfa3-1* (Salk_011107)^38^, *dreb2a-1* (379F02 GABI-KAT)^39^ and *hsp101*^23^ mutants have been described. Seedlings were grown on GM medium (1% [w/v] glucose) under a 16 h/8 h light/dark cycle at 23/21 °C^23^. Primer sequences for genotyping are listed in Supplementary Table 4.

### HS treatments

4 d-old seedlings were exposed to 44 °C for 25–45 min to examine basal thermotolerance (bTT); to examine acquired thermotolerance (aTT) they were exposed to 37 °C for 1 h, 23 °C for 90 min and 44 °C for 160–250 min^23^. For HS memory assays, 4 d-old seedlings were primed with a two-step acclimation (ACC) protocol consisting of 37 °C for 1 h, 23 °C for 90 min and 44 °C for 45 min^23^. Primed seedlings were exposed to a triggering HS at 44 °C for 70–130 min on day 7 or 44 °C for 30–80 min on day 8–10.

### Construction of *HSFA3* complementation and promoter swap constructs

To obtain a genomic fragment containing the *HSFA3* gene as well as flanking regions until the borders of the neighboring genes, PCR with primers 2410 containing an *Asc*I site and 2418 containing a *Pac*I site was performed and the resulting product was subcloned into pJET1.2 (Thermo Fisher). After sequencing, the genomic *HSFA3* fragment was introduced using *Asc*I and *Pac*I into a *pGreenII* binary vector harboring a Norflurazone resistance (kindly provided by T. Laux). To obtain a FLAG-tagged version of *HSFA3* driven by the native promoter the promoter flanked by *Asc*I and *Age*I (primers 2410 and 2420) and *3xFLAG-HSFA3* flanked by *Age*I until the beginning of the downstream neighboring gene flanked by a *Pac*I-site (Primers 2417 and 2418) were amplified and the resulting fragments subcloned into pJET1.2. After sequencing, the two fragments were combined in pJET1.2 via *Age*I and *Pac*I and the final fragment introduced into *pGreenII* with Norflurazone resistance. In order to generate promoter swap constructs, either *pHSFA2* (primers 2624/2625), *HSFA2-YFP* (primers 2786/2787), or *3xFLAG-HSFA1D* (primers 2810/2811) were amplified, subcloned into *pJET1.2* and sequenced. *pHSFA2* replaced *pHSFA3* and *HSFA2-YFP* or *3xFLAG-HSFA1D* replaced *3xFLAG-HSFA3* in the *pHSFA3::3xFLAG-HSFA3* construct to obtain *pHSFA2::3xFLAG-HSFA3*, *pHSFA3::HSFA2-YFP* and *pHSFA3::3xFLAG-HSFA1D*. All constructs were introduced into the *GV3101* strain of *Agrobacterium tumefaciens* and transformed using the floral dip method^55^. Primer sequences are listed in Supplementary Table 4.

### RNA extraction and qRT-PCR

To examine sustained induction of gene expression, 4 d-old seedlings were exposed to an ACC treatment and samples were taken during the following 3 d as indicated. To study enhanced re-induction of memory genes, seedlings were treated for 1 h at 37 °C on day four and again on day six or day seven as indicated. Samples including non-treated controls were taken at the end of the last HS. RNA was extracted from seedlings using hot-phenol RNA extraction: frozen tissue was ground to a powder and resuspended in 500 µl homogenization buffer (100 mM Tris pH 8.0, 5 mM EDTA, 100 mM NaCl, 0.5% SDS), 250 µl phenol and 5 µl ß-mercaptoethanol and incubated for 15 min at 60 °C. 250 µl chloroform was added and samples were incubated for 15 min at 60 °C before spinning 10 min at 13000 rpm. 550 µl aqueous phase was transferred into a new tube containing 550 ul phenol:chloroform:isoamylalcohol (25:24:1), mixed and centrifuged as above. 500 µl aqueous phase was transferred into a new tube containing 50 µl 3 M sodium acetate and 400 µl isopropanol and precipitated at −80 °C. After 30 min of centrifugation at 4 °C and 13000 rpm, pellets were dried and resuspended in 500 µl H_2_O. 500 µl 4 M LiCl was added and RNA was precipitated overnight at 4 °C. RNA was pelleted, washed in 80% EtOH, dried, and resuspended in 40 µl H_2_O. For quantitative RT-PCR, total RNA was treated with TURBO DNA-free (Ambion) and reverse transcribed with SuperScript III (Invitrogen) according to manufacturers instructions. 0.1 μl cDNA was used per 10 μl QPCR reaction with GoTaq qPCR Master Mix (Promega) and LightCycler 480 (Roche)^23^. All data were normalized to the reference gene *At4g26410* ^56^ using the comparative CT method. Primers are listed in Supplementary Table 4.

### RNA-seq

For RNA-seq analysis, total RNA was extracted from Col-0, *hsfa2*, *hsfa3-1*, and *hsfa2, 3-1* seedlings with RNeasy Plant Mini kit (Qiagen). On-column DNase digest of RNA was performed with RNase-Free DNase (Qiagen). RNA quality control, library preparation, and sequencing were performed by BGI Genomics (http://www.bgi.com) with the DNBseq platform generating 2 × 150 bp paired-end sequencing reads. Three biological replicates were generated and analyzed per treatment and genotype. Reads were mapped against the *Arabidopsis thaliana* reference genome (TAIR10) using STAR^57^ version 2.5.1a. Quantification at gene level was done using STAR with the quantMode GeneCounts option. Differential gene expression analysis was done using the R (https://www.r-project.org) package DESeq2^58^. Only protein-coding genes were analyzed, transposable element genes were excluded. Col 1-1-1 up genes were defined as being induced above baseline non-heat stressed level (defined as log_2_(fold change)>1, *p* < 0.05) at 4 h, 28 h, and 52 h after ACC treatment. 156 such genes were identified in the Col-0 background. Col 1-0-0 up genes were defined as being induced above baseline non-heat stressed level (defined as log_2_(fold change)>1, *p* < 0.05) at 4 h after ACC treatment, but not at 28 h or 52 h after ACC treatment. 3225 such genes were identified in the Col-0 background. Genes were counted as “not upregulated in mutant” at 4 h, 28 h, or 52 h after ACC treatment relative to no-HS level when either one of the following conditions was met: log_2_(fold change)≤1 or log_2_(fold change)>1, *p* > 0.05. Data visualizations were done using the R package ggplot2 (https://ggplot2.tidyverse.org).

### Chromatin immunoprecipitation

All heat-treated samples were exposed to an ACC treatment, seedlings harvested at the indicated time points after ACC and cross‐linked under vacuum in ice‐cold MC buffer/ 1% (v/v) formaldehyde^59^ for 2 × 5 min for histone ChIP or 2 × 10 min for 3xFLAG-HSFA2 or 3xFLAG-HSFA3 ChIP. Chromatin was extracted as follows;^59^ frozen tissue was ground up and resuspended in 25 ml M1 buffer, washed five times in 5 ml M2 buffer, and once in 5 ml M3 buffer with centrifugation for 10 min, 1000 g for each washing step. The resulting chromatin pellet was taken up in 1 ml of sonication buffer (buffer recipes described in^59^). Chromatin was sonified using a Diagenode Bioruptor (17 cycles/30 sec on/off) on low-intensity settings. For histone ChIP, equal amounts of chromatin from the same preparation were immunoprecipitated at 4 °C overnight using antibodies against H3 (ab1791, Abcam) or H3K4me3 (ab8580, Abcam). For 3xFLAG-HSFA2/3-ChIP, chromatin was incubated with anti‐DYKDDDDK paramagnetic beads for 1 h at 4 °C and chromatin was recovered using a DYKDDDDK isolation kit (both Miltenyi Biotec). Immunoprecipitated DNA was quantified by qPCR (LightCycler480, Roche).

### Yeast-two-hybrid analysis

*HSFA2* and *HSFA3* full and truncated (without AHA domains) coding regions were amplified and inserted into pGBKT7 and pGADT7 (Clontech), through either Gateway® technology (Invitrogen) or restriction enzyme (*BamH*I and *EcoR*I) digestion. Yeast cultures (MaV203 strain) were grown at 28 °C on Yeast Peptone Dextrose (YPD) or Synthetic Dextrose (SD) media supplemented with selective Drop-out (DO) aminoacid mixtures. Double transformation with both pGBKT7 (bait) and pGADT7 (prey) constructs was performed according to standard protocols. Transformants were selected on SD medium supplemented with DO–Trp–Leu (SD–WL) and protein interaction was analyzed by growth on SD medium supplemented with DO–Trp–Leu–His (SD–WLH) and 50 mM 3-Amino-1,2,4-triazole (3-AT).

### Co-IP, immunoblotting, and mass spectrometry

Total native protein complexes were isolated from 1 g of seedlings in 4 ml of Extraction buffer (50 mM Tris–HCl pH7.5, 150 mM NaCl, 2% Triton X-100, 1 Tablet complete mini Protease inhibitor cocktail (Roche)/25 ml) and centrifuged 4 times at 4 °C and maximum speed for 10 min. 100 µl input was taken from the supernatant and 2 ml protein extract were incubated with 50 µl of α‐DYKDDDDK paramagnetic beads for 1.5 h at 4 °C. Protein complexes were recovered using a DYKDDDDK isolation kit (Miltenyi Biotec) and 3 washes with wash buffer (50 mM Tris–HCl pH7.5, 150 mM NaCl, 0.1% Triton X-100, 1 Tablet complete mini Protease inhibitor cocktail (Roche) /25 ml). For mass spectrometry, 3 more washes with 20 mM Tris–HCl were performed. Proteins were eluted in 50 µl 8 M urea and used for immunoblotting^32^ with anti‐GFP (ab290, Abcam, 1:2000), anti‐FLAG (M2, F1804, Sigma, 1:2500), anti H3 (ab1791, abcam, 1:5000) or anti‐Tubulin (T5168, Sigma, 1:4000) antibodies. For mass spectrometry, eluates were further processed as described^24^. Briefly, eluates were diluted and digested with Trypsin (Fig. 7) or Trypsin/Lys-C Mix (Supplementary Table 3, Promega). Peptides were desalted, lyophilized and re-suspended in 20 μl 3% (v/v) acetonitrile, 0.1% (v/v) formic acid. Measurements were performed on a Q Exactive Plus Orbitrap mass spectrometer coupled with an Easy nLC1000 HPLC (Thermo Fisher Scientific, Fig. 7) a Q Exactive HF (Thermo Fisher Scientific) mass spectrometer coupled with an Aquity M class UPLC (Waters, Supplementary Table 3).

### In vitro pulldown assay

Coding sequences of HSFA proteins were inserted into the pIX-HALO expression vector by Gateway cloning. Primers are listed in Supplementary Table 4. For HSFA2 and HSFA3, the Halo tag was replaced by a 3xFLAG tag to yield pIX-FLAG expression vectors. For each pulldown reaction, 500 ng of each plasmid were mixed and transcription and translation were carried out in TNT wheat germ expression kits (Promega) according to manufacturer’s instructions. Proteins were incubated overnight with Magne HaloTag beads (Promega), washed three times in PBS/ 0.1% NP-40, and eluted in SDS loading buffer. Samples were analyzed by SDS-page and immunoblotting using anti-FLAG (M2, F1804, Sigma, 1:2500) and monoclonal anti-Halo (G9211, Promega, 1:2000) antibodies.

### Promoter analysis

The sequences for HSFA2 and HSFA3 promoters were analyzed using JASPAR^60^. The profiles for Arabidopsis HSF (MA1664.1, MA1665.1, MA1666.1, MA1667.1) and DREB2 (MA0986.1, MA1258.1) binding sites were selected for promoter analysis and analyzed with standard settings (profile score threshold 80%).

### Reporting Summary

Further information on research design is available in the Nature Research Reporting Summary linked to this article.

## Supplementary information

Supplementary Information

Descriptions of Additional Supplementary Files

Supplementary Data 1

Reporting Summary

## Data Availability

RNA sequencing data have been deposited at NCBI GEO under accession number GSE162434. All raw data underlying the individual figures are provided as Supplementary Data. The plant materials generated and analyzed during the current study are available from the corresponding author upon request. Source data are provided with this paper.

## Source data

Source Data
