# Supplementary material for: Heteromeric HSFA2/HSFA3 complexes drive transcriptional memory after heat stress in Arabidopsis
Source: Nat Commun. 2021 Jun 8;12:3426. doi: 10.1038/s41467-021-23786-6 (PMC8187452; doi:10.1038/s41467-021-23786-6)
Supplement: Supplementary file 1 — Supplementary Information [file 41467_2021_23786_MOESM1_ESM.pdf]

Heteromeric HSFA2/HSFA3 complexes drive transcriptional memory after heat stress in *Arabidopsis*

Thomas Friedrich[1], Vicky Oberkofler[1], Inês Trindade[1], Simone Altmann[1+], Krzysztof Brzezinka[1], Jörn Lämke[1], Michal Gorka[2], Christian Kappel[1], Ewelina Sokolowska[2], Aleksandra Skirycz[2], Alexander Graf[2], Isabel Bäurle[1]

Supplementary Figures and Tables

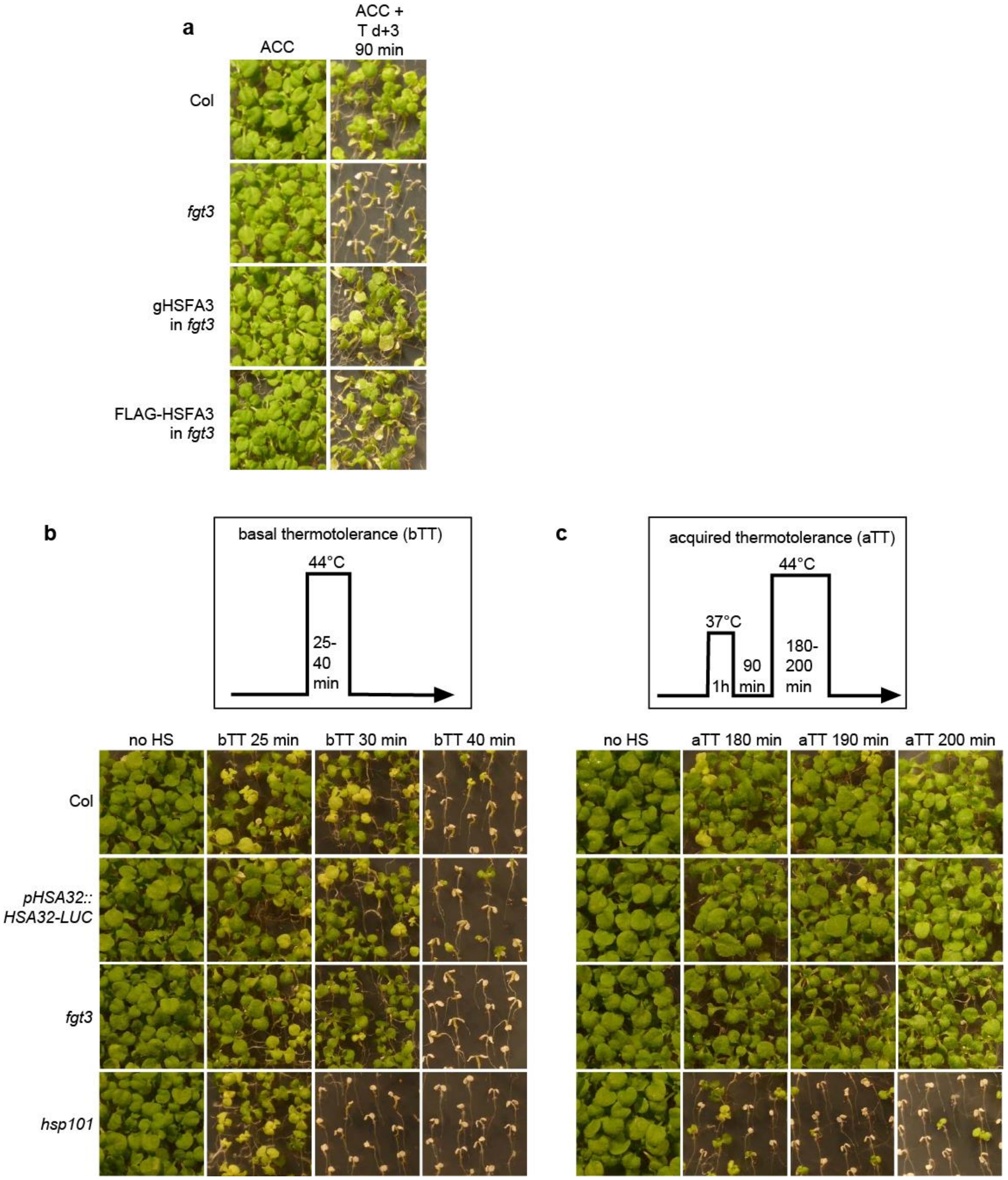

Supplementary Fig. 1 *FGT3* is required for HS memory, but not basal thermotolerance or acquired thermotolerance

a-c Characterization of different HS responses in *fgt3* mutants.

a *fgt3* is defective in physiological HS memory. 4 d-old seedlings were exposed to an ACC treatment and exposed to a triggering HS (T) at 44°C for 90 min on d+3 after ACC (cf. Fig. 2a). The defect was complemented by transformation with a genomic *HSFA3* fragment (*gHSFA3*) or by *pHSFA3::FLAG-HSFA3* (*FLAG-HSFA3*). Images were taken 14 d after ACC.

b Basal thermotolerance is not affected in *fgt3* mutants. Treatment scheme for basal thermotolerance (bTT) assays (Stief et al., 2014). 4 d-old seedlings were exposed to 44°C for 25-40 min and images were taken 14 d later. The *hsp101* mutant is shown as a control for decreased basal thermotolerance.

c Acquired thermotolerance is not affected in *fgt3* mutants. Treatment scheme for acquired thermotolerance (aTT) assays (Stief et al., 2014). 4 d-old seedlings were exposed to 37°C for 1 h, recovered at 23°C for 90 min and subsequently exposed to 44°C for 180-200 min. Images were taken 14 d later. The *hsp101* mutant is shown as a control for decreased acquired thermotolerance.

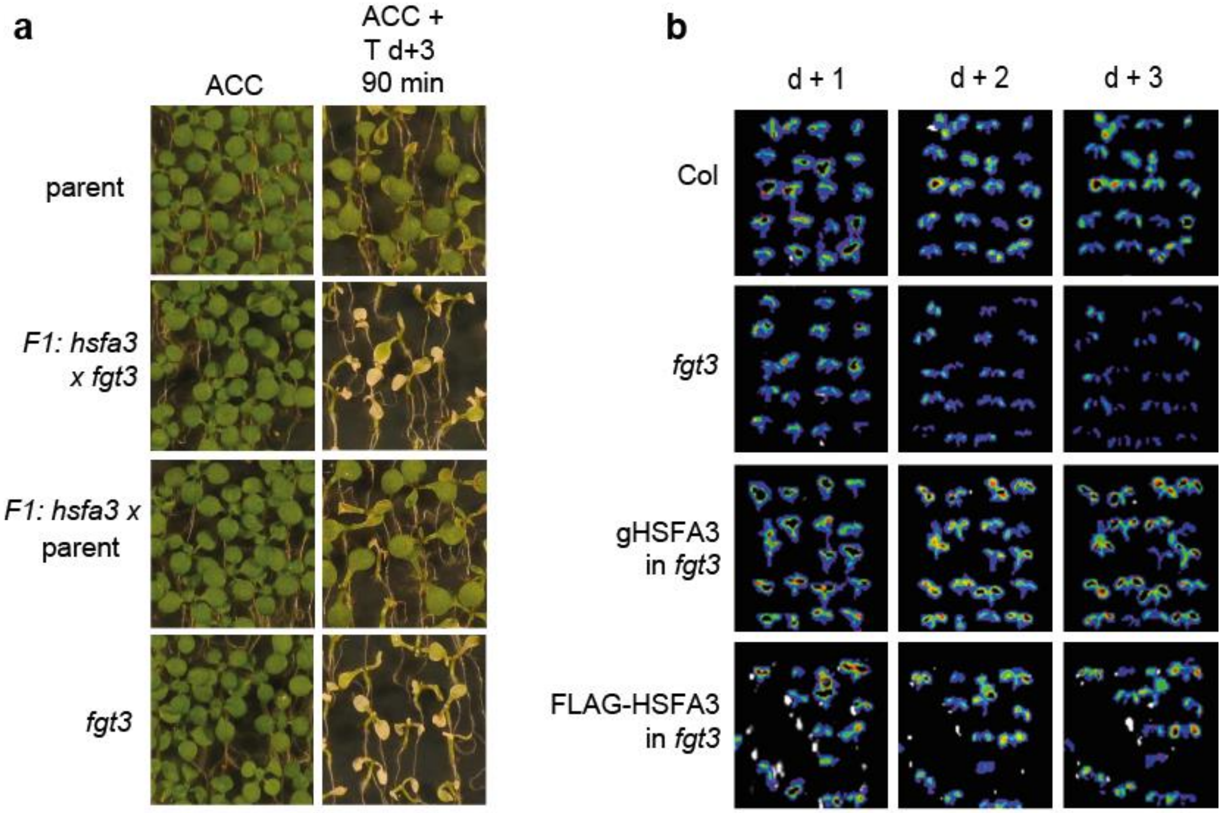

Supplementary Fig. 2 *FGT3* encodes *HSFA3*

a Complementation crossing for *hsfa3-1* and *fgt3*; like *fgt3* mutants, the F1 progeny of *hsfa3-1* x *fgt3* showed decreased survival in HS memory assays compared to the parental *pHSA32::HSA32-LUC* line or the F1 progeny of *hsfa3-1* x *pHSA32::HSA32-LUC*. 4 d-old seedlings were exposed to an ACC treatment and then to a triggering HS (T) at 44°C for 90 min on d 3 after ACC (cf. Fig. 2a). Images were taken 14 d after ACC.

b *fgt3* mutants are complemented by either a genomic *HSFA3* fragment (*gHSFA3*) or a *pHSFA3::FLAG-HSFA3* construct (*FLAG-HSFA3*) in sustained induction of *HSA32-LUC* expression. 4 d-old seedlings were exposed to an ACC treatment and activity of *pHSA32::HSA32-LUC* was recorded for the 3 following days (cf. Fig. 1a).

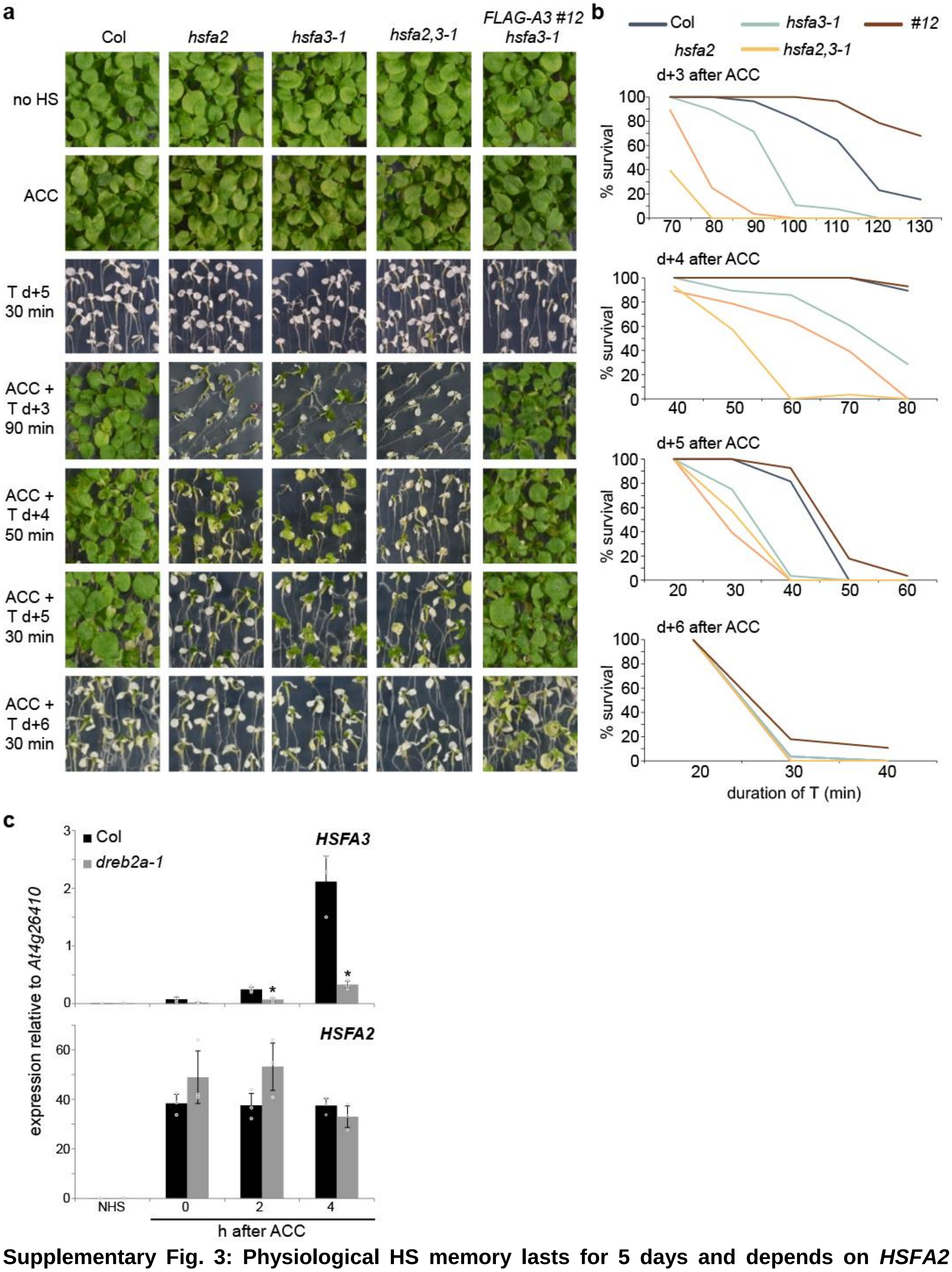

Supplementary Fig. 3: Physiological HS memory lasts for 5 days and depends on *HSFA2* and *HSFA3*

Long term HS memory assay for *hsfa2*, *hsfa3-1*, *hsfa2 hsfa3-1* mutants and the native *FLAG-HSFA3* overexpressing line #12.

a Representative images of survival assays with plants exposed to triggering HS (T) of different durations on d +3, 4, 5 and 6 after ACC. Naïve plants were unable to survive a triggering HS of 30 min. Up to 5 d after ACC, primed plants survived triggering HS of 30 min or longer; by d 6 after ACC, HS memory was no longer detectable in Col.
b Percentage of survival of the genotypes tested in (**a**) across all tested time points on d +3, 4, 5, and 6 after ACC.
c *HSFA3* induction after ACC is reduced in *dreb2a-1* mutants. Transcript levels of *HSFA3* and *HSFA2* relative to *At4g26410* reference gene in Col and *dreb2a-1* mutants at the indicated time after ACC treatment or NHS as measured by qRT-PCR. Data are mean ± SD of three independent experiments. Asterisks mark significant differences to Col (unpaired two-sided t-test, $p<0.01$).

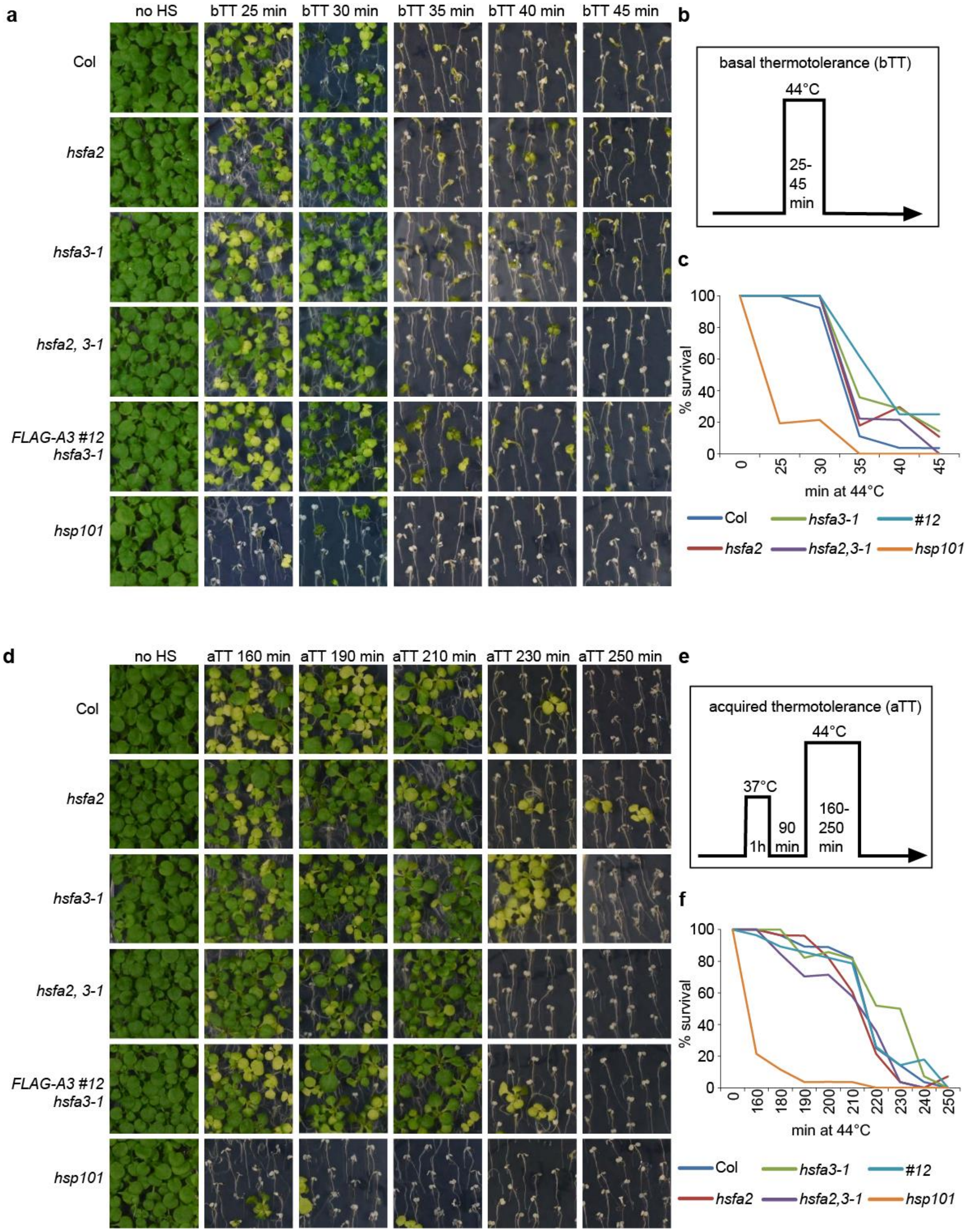

Supplementary Fig. 4: HSFA2 and HSFA3 are not required for basal or acquired thermotolerance

a-c Basal thermotolerance assay for *hsfa2*, *hsfa3*, *hsfa2 hsfa3-1* mutants and *FLAG-HSFA3* native overexpressing line #12. **b** Treatment scheme for basal thermotolerance (bTT) assays: 4 d-old seedlings were exposed to 44°C for 25-45 min and survival was scored 14 d later. **c** Percentage of surviving plants after bTT assay.

d-f Acquired thermotolerance assay for *hsfa2*, *hsfa3*, *hsfa2 hsfa3-1* mutants and *FLAG-HSFA3* native overexpressing line #12. **e** Treatment scheme for acquired thermotolerance (aTT) assays: 4 d-old seedlings were exposed to 37°C for 1 h, recovered at 23°C for 90 min and subsequently exposed to 44°C for 160-250 min. Survival was scored 14 d later. **f** Percentage of surviving plants after aTT assay. The *hsp101* mutant is shown as a control for decreased aTT and bTT.

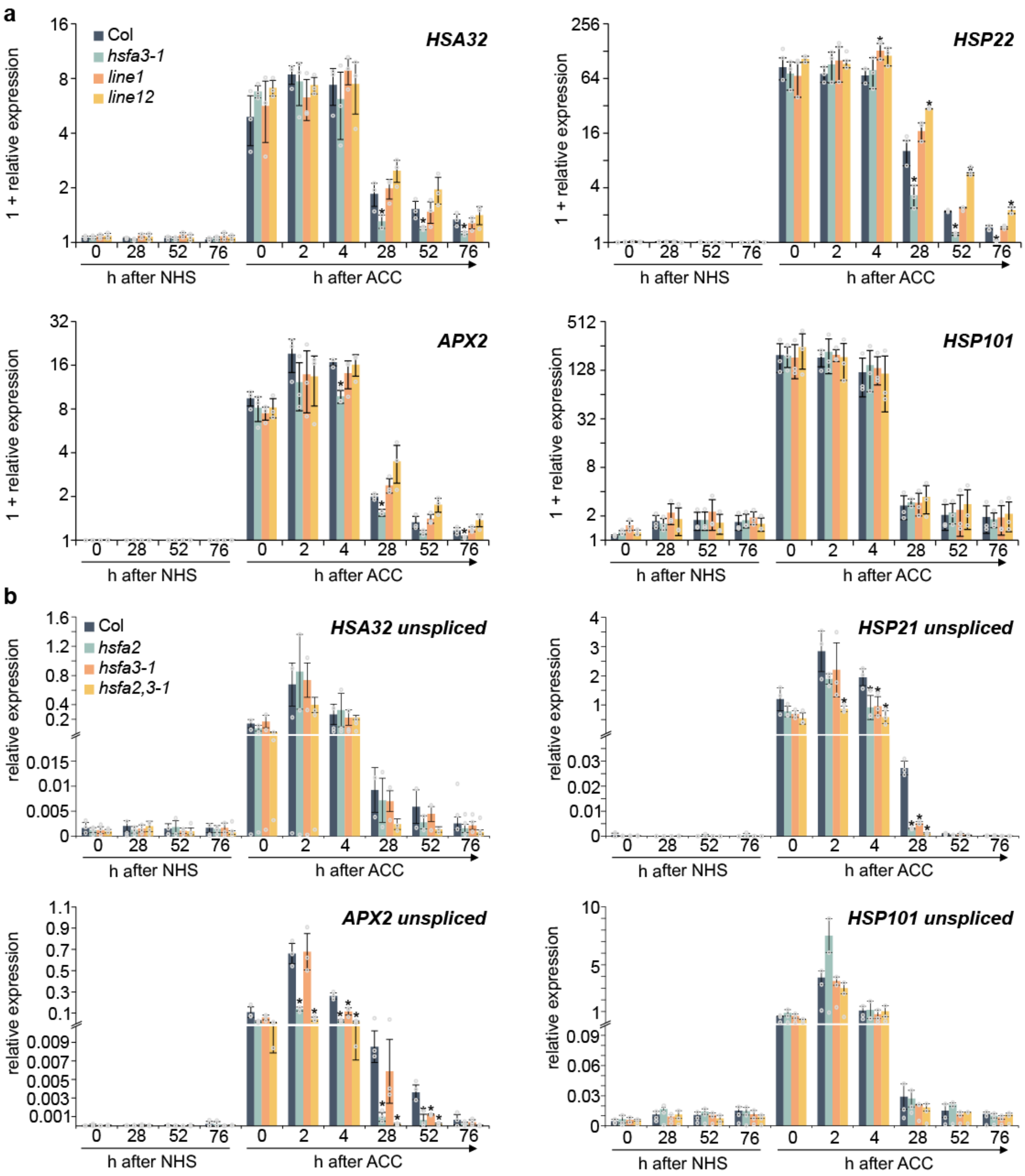

Supplementary Fig. 5 *pHSFA3::FLAG*-HSFA3 rescues the sustained induction defects of memory genes in *hsfa3-1* and sustained induction of unspliced transcripts in mutants

a Relative transcript levels of the memory genes *HSA32*, *HSP22* and *APX2* and the non-memory gene *HSP101* as measured by qRT-PCR.

b Relative levels of unspliced transcripts of the memory genes *APX2*, *HSA32* and the non-memory gene *HSP101* as measured by qRT-PCR.

Time points depict h after ACC treatment. Data are mean ± SD of three independent experiments. Asterisks mark significant differences to Col ($p<0.05$, unpaired two-sided t-test).

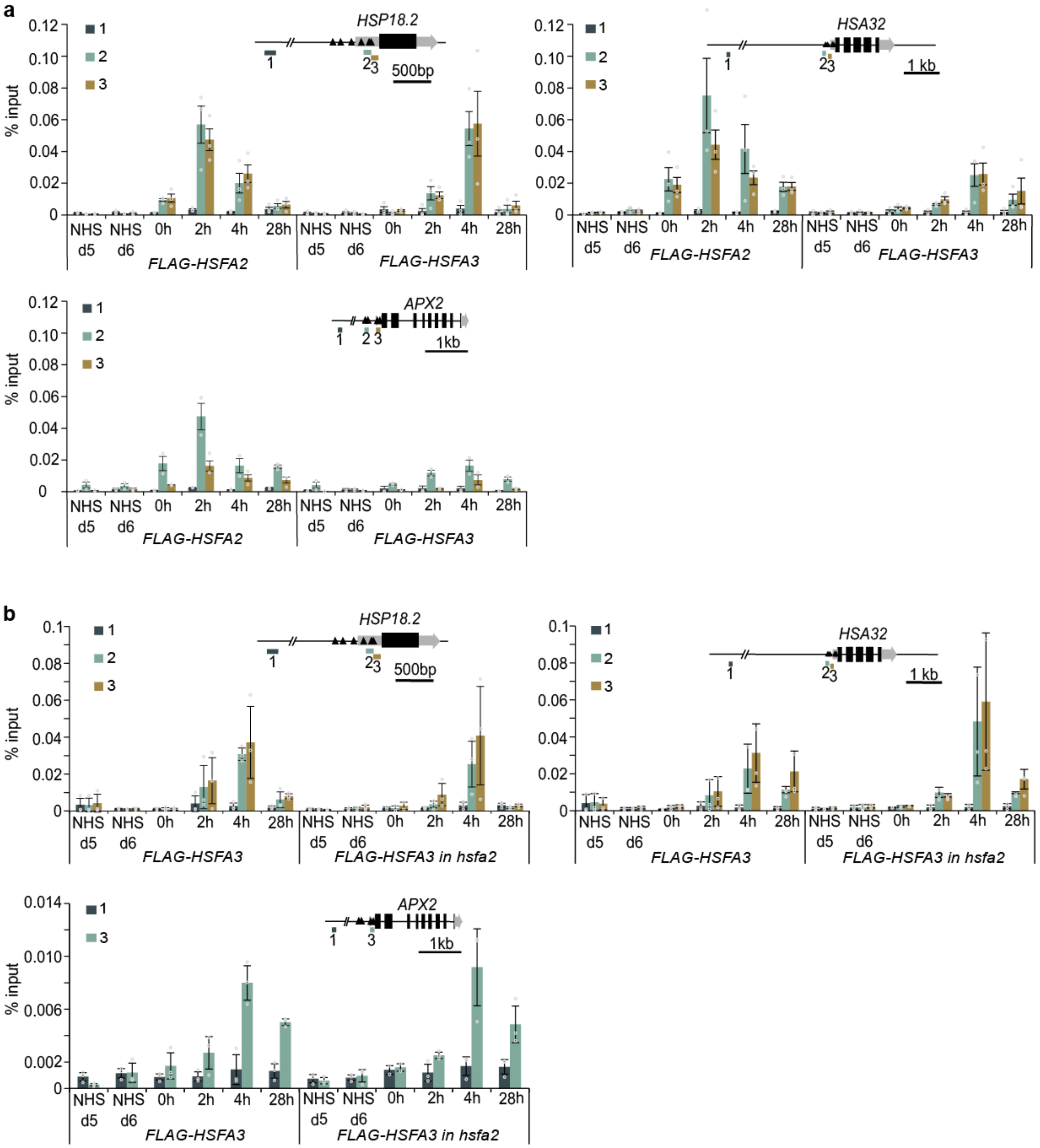

Supplementary Fig. 6 HSFA2 and HSFA3 bind to memory gene promoters (additional target genes, cf. Fig. 8)

a Occupancy of HSFA2 and HSFA3 as determined by ChIP-qPCR from *pHSFA2::FLAG-HSFA2* and *pHSFA3::FLAG-HSFA3*.

b Occupancy of HSFA3 as determined by ChIP-qPCR from *pHSFA3::FLAG-HSFA3* in the wild type or *hsfa2* mutant background.

Enrichment normalized to Input from three independent experiments for the HS memory genes *HSP18.2*, *HSA32*, and *APX2*. Time points are given in h after ACC treatment. Data are mean ± SD. For each locus one control amplicon situated approximately 3 kb upstream is shown alongside the amplicon covering heat shock elements (black triangles) in the promoter (inset gene models).

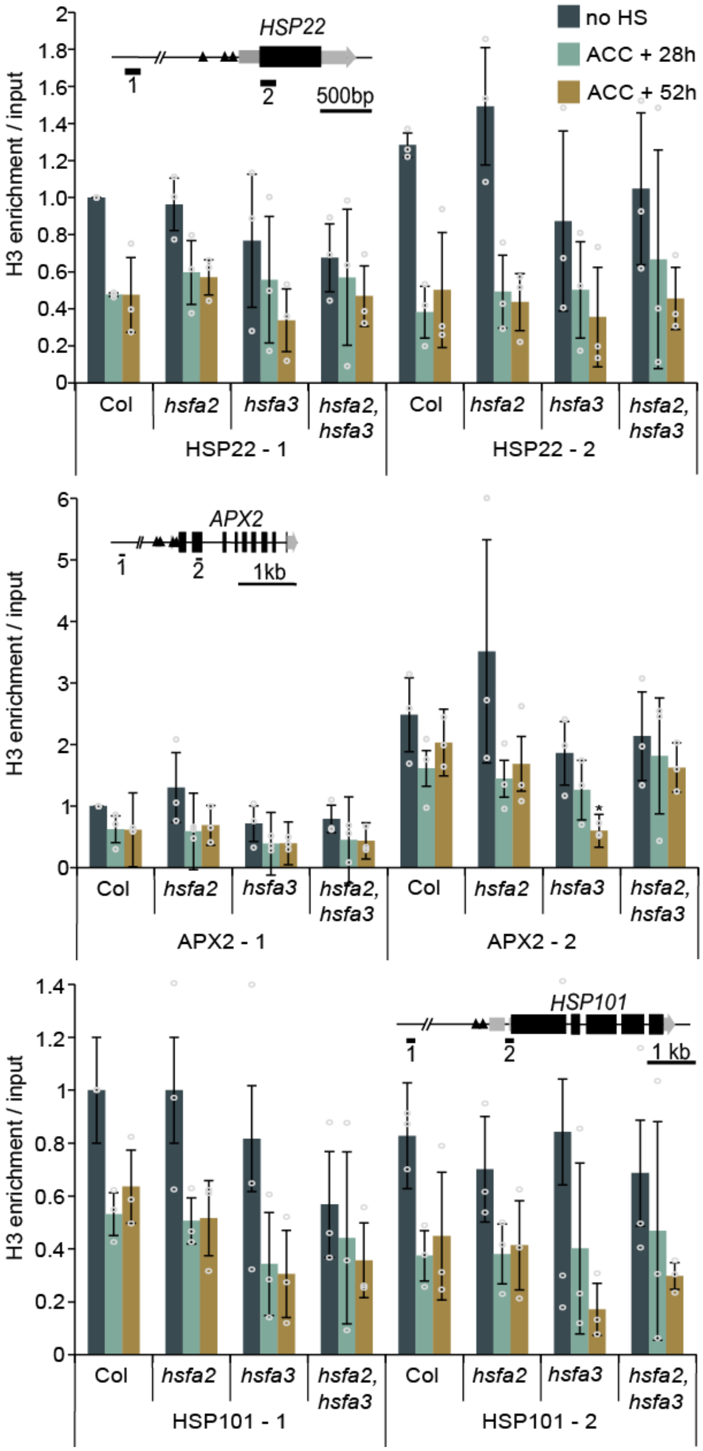

Supplementary Fig. 7 Histone H3 enrichment after ACC (cf. Fig. 9)

Enrichment of histone H3 relative to input for the HS memory genes *HSP22* and *APX2*, and the non-memory gene *HSP101* as determined by ChIP-qPCR from three independent experiments. Data were normalized to Col amplicon 1 for each gene. For each locus the control amplicon 1 situated approximately 3 kb upstream is shown alongside the amplicon covering the transcriptional start site (inset gene models). Time points are given in h after end of ACC treatment. Data are mean ± SD. Asterisks mark significant differences to Col (p<0.01, unpaired two-sided t-test).

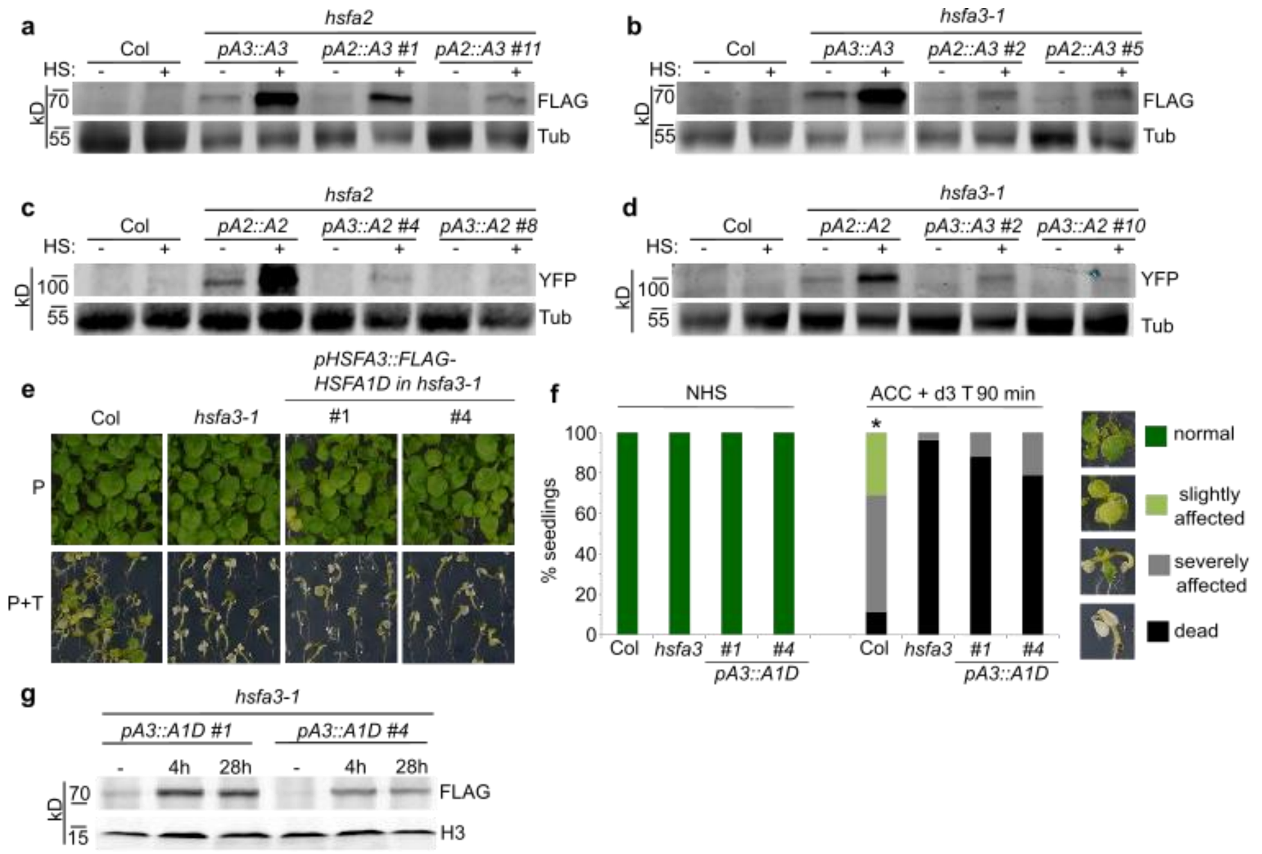

Supplementary Fig. 8 Protein expression of promoter-swap constructs (cf. Fig. 10) and HSFA1D protein cannot functionally replace HSFA3

a-d Expression of FLAG-HSFA3 or HSFA2-YFP from *pHSFA2::FLAG-HSFA3* (**a-b**) or *pHSFA3::HSFA2-YFP* (**c-d**) in *hsfa2* (**a**,**c**) or *hsfa3-1* mutants (**b**,**d**), respectively, as shown by immunoblotting with the indicated antibodies.

e HS memory assay for *hsfa3-1* and two independent transgenic lines expressing FLAG-HSFA1D from the *HSFA3* promoter. 4 d-old seedlings were exposed to an ACC treatment and to a triggering HS (T) at 44°C 3 d after ACC (P+T).

f Distribution of phenotypic categories observed in the HS memory assay shown in **e**. Asterisk depicts significant differences to *hsfa3-1* mutants (p=0.0000019, Fisher's exact test, n≥20 seedlings per genotype).

g Protein expression of FLAG-HSFA1D from *pHSFA3::FLAG-HSFA1D* in the absence of HS treatment (-) or 4-28 h after ACC as shown by immunoblotting with the indicated antibodies.

Protein expression experiments (**a, b, c, d, g**) were repeated 3 times with similar results.

Supplementary Table 1: HSF- and DREB-binding motifs in *HSFA2* and *HSFA3* promoters identified by JASPAR (http://jaspar.genereg.net/)

Matrix ID	Name	Score	Relative score	Sequence ID	Start	End	Strand	Predicted sequence
MA1665.1	**HSFB2A**	18.5785	0.983397096	**HSFA2**	500	511	-	gaaggttctaga
MA1667.1	**HSFC1**	17.4454	0.98016547	**HSFA2**	501	511	+	ctagaaccttc
MA1667.1	**HSFC1**	15.5783	0.950466801	**HSFA2**	410	420	-	ccagaaacttc
MA1665.1	**HSFB2A**	15.9492	0.940603946	**HSFA2**	410	421	+	gaagtttctgga
MA1664.1	**HSFA6B**	18.1615	0.939249994	**HSFA2**	498	512	-	agaaggttctagaga
MA1666.1	**HSFB2B**	18.6039	0.938907035	**HSFA2**	499	512	+	ctctagaaccttct
MA1664.1	**HSFA6B**	16.6796	0.916784202	**HSFA2**	409	423	+	cgaagtttctggaac
MA1666.1	**HSFB2B**	16.6508	0.91166361	**HSFA2**	409	422	-	ttccagaaacttcg
MA1665.1	**HSFB2A**	11.2508	0.864134403	**HSFA2**	465	476	+	gaagcttcaata
MA1666.1	**HSFB2B**	12.7155	0.8567711	**HSFA2**	314	327	-	ttctaggtccttct
MA1664.1	**HSFA6B**	12.6872	0.856261914	**HSFA2**	314	328	+	agaaggacctagaac
MA0986.1	**DREB2C**	7.36964	0.852137169	**HSFA2**	296	303	-	ggccgcca
MA1665.1	**HSFB2A**	9.47322	0.835202346	**HSFA2**	461	472	-	gaagcttctttg
MA1666.1	**HSFB2B**	11.1379	0.834766401	**HSFA2**	415	428	+	ttctggaacattgt
MA1665.1	**HSFB2A**	9.34876	0.83317656	**HSFA2**	416	427	-	caatgttccaga
MA1664.1	**HSFA6B**	10.6675	0.825645298	**HSFA2**	414	428	-	acaatgttccagaaa
MA1665.1	**HSFB2A**	8.57313	0.820552734	**HSFA2**	315	326	+	gaaggacctaga
MA1667.1	**HSFC1**	7.37575	0.819991162	**HSFA2**	315	325	-	ctaggtccttc
MA1664.1	**HSFA6B**	9.77443	0.812106708	**HSFA2**	658	672	-	gtaactttcgagaaa
MA1665.1	**HSFB2A**	8.0052	0.811309289	**HSFA2**	390	401	+	agagattctaga
MA1667.1	**HSFC1**	6.62239	0.808007658	**HSFA2**	57	67	-	cgaggaacttc
MA0986.1	**DREB2C**	5.31175	0.807983832	**HSFA2**	227	234	-	gacctaca
MA1666.1	**HSFB2B**	9.03637	0.805452774	**HSFA2**	659	672	+	ttctcgaaagttac
MA1665.1	**HSFB2A**	7.642	0.805397953	**HSFA2**	660	671	-	taactttcgaga
MA1665.1	**HSFB2A**	7.62676	0.805149815	**HSFA2**	406	417	-	gaaacttcgtta

Matrix ID	Name	Score	Relative score	Sequence ID	Start	End	Strand	Predicted sequence
MA0986.1	**DREB2C**	11.5636	0.942120189	**HSFA3**	969	976	-	aaccgaca
MA0986.1	**DREB2C**	11.5636	0.942120189	**HSFA3**	1136	1143	-	aaccgaca
MA0986.1	**DREB2C**	9.31686	0.893915977	**HSFA3**	1088	1095	+	caccgcct
MA1258.1	**DREB2**	14.3269	0.890856893	**HSFA3**	1081	1101	-	atagagaggcggtggaaatgg
MA1258.1	**DREB2**	12.8104	0.868889881	**HSFA3**	963	983	+	tttcaatgtcggttgtacaat
MA1665.1	**HSFB2A**	9.90829	0.842283367	**HSFA3**	995	1006	+	aaagtttccaaa
MA0986.1	**DREB2C**	5.60096	0.814188997	**HSFA3**	519	526	+	aaccaaca
MA1665.1	**HSFB2A**	7.68134	0.806038239	**HSFA3**	608	619	+	aaaatatctaga

Supplementary Table 2: Unique peptide numbers of HSF-proteins identified in IP-MS experiments

Plants were exposed to either 37°C for 1h or a full ACC treatment and samples were taken at the indicated times. Unique peptide numbers from three independent experiments are shown for each genotype and condition.

**HSFA2-YFP IP-MS**													
		**Col**			**NHS**			**37°C + 45min**			**37°C + 3h**		
		**1**	**2**	**3**	**1**	**2**	**3**	**1**	**2**	**3**	**1**	**2**	**3**
AT2G26150	HSFA2	3	0	0	23	23	25	22	27	26	26	27	28
AT5G03720	HSFA3	0	0	0	1	0	2	2	9	2	6	9	11
AT4G17750	HSFA1A	0	0	0	4	4	5	2	6	4	4	6	5
AT5G16820	HSFA1B	0	0	0	4	5	5	5	10	5	6	10	7
AT1G32330	HSFA1D	0	0	0	6	6	8	5	9	7	7	9	7
AT3G22830	HSFA6B	0	0	0	1	1	3	0	6	1	1	6	7
AT3G51910	HSFA7A	0	0	0	0	0	0	7	11	11	9	10	13
AT3G63350	HSFA7B	0	0	0	0	0	0	0	4	0	1	4	5

**3xFLAG-HSFA3 IP-MS**													
		**Col**			**NHS**			**37°C + 4h**			**ACC + 4h**		
		**1**	**2**	**3**	**1**	**2**	**3**	**1**	**2**	**3**	**1**	**2**	**3**
AT5G03720	HSFA3	0	1	2	8	10	10	14	14	14	16	17	18
AT2G26150	HSFA2	1	1	1	0	1	1	11	9	11	14	13	13
AT1G32330	HSFA1D	0	0	0	3	3	4	4	6	3	5	7	6
AT5G16820	HSFA1B	0	0	0	1	2	0	3	5	3	6	6	6
AT3G22830	HSFA6B	0	0	0	0	1	0	0	1	1	2	5	5
AT3G51910	HSFA7A	0	0	0	0	0	0	2	2	2	2	3	3
AT4G17750	HSFA1A	0	0	0	0	0	0	0	0	0	1	1	1

Supplementary Table 3: Unique peptide numbers of HSF-proteins identified by Co-IP/MS experiments of 3xFLAG-HSFA2, 3xFLAG-HSFA3 in wild type and *hsfa* mutant backgrounds

Plants were exposed to ACC treatment and harvested 4 h after the end of the treatment. Protein complexes were purified with anti-FLAG in all samples.The number of unique peptides is displayed for five independent biological replicates per genotype x treatment.

	3xFLAG-HSFA2										3xFLAG-HSFA2 in *hsfa3*										Col									
	NHS					ACC +4h					NHS					ACC +4h					NHS					ACC +4h				
replicate	1	2	3	4	5	1	2	3	4	5	1	2	3	4	5	1	2	3	4	5	1	2	3	4	5	1	2	3	4	5
HSFA3	-	-	-	-	7	-	3	2	4	3	-	-	-	-	-	-	-	-	2	-	-	-	-	1	-	-	-	-	-	-
HSFA2	-	1	2	6	1	5	13	9	14	10	1	-	1	-	-	14	6	19	20	6	-	-	1	1	-	1	1	1	-	-
HSFA1D	-	-	-	1	2	-	1	1	1	1	-	-	-	-	-	3	2	5	8	-	-	-	-	-	-	-	-	-	-	-
HSFA1B	-	-	-	-	-	-	3	-	3	1	-	-	-	-	-	4	3	5	6	2	-	-	-	1	-	-	-	-	-	-
HSFA1A	-	-	-	-	1	-	-	-	-	-	-	-	-	-	-	-	-	-	4	-	-	-	-	-	-	1	-	-	-	-
HSFA7B	-	-	-	-	-	-	-	-	1	-	-	-	-	-	-	-	-	3	3	-	-	-	-	-	-	-	-	-	-	-
HSFA7A	-	-	-	-	-	-	-	-	-	-	-	-	-	-	-	-	-	-	-	-	-	-	-	-	-	-	-	-	-	-
HSFA6B	-	-	-	-	-	-	-	-	-	-	-	-	-	-	-	-	-	-	-	-	-	-	-	-	-	-	-	-	-	-

	3xFLAG-HSFA3										3xFLAG-HSFA3 in *hsfa2*									
	NHS					ACC +4h					NHS					ACC +4h				
replicate	1	2	3	4	5	1	2	3	4	5	1	2	3	4	5	1	2	3	4	5
HSFA3	6	2	4	4	11	18	19	5	20	18	11	4	5	12	1	20	7	9	20	9
HSFA2	-	1	-	2	1	12	16	4	18	16	-	-	1	1	4	-	-	-	1	-
HSFA1D	1	-	1	1	5	4	8	-	8	6	2	2	1	4	1	6	1	3	8	1
HSFA1B	1	-	-	-	-	3	5	1	6	5	1	1	-	2	-	5	3	4	6	2
HSFA1A	1	-	-	-	1	-	2	-	2	1	1	-	-	1	-	4	-	-	4	-
HSFA7B	-	-	-	-	-	-	-	-	-	-	-	-	-	-	-	-	-	-	-	-
HSFA7A	-	-	-	-	-	-	-	-	-	-	-	-	-	-	-	-	-	-	-	-
HSFA6B	-	-	-	-	-	-	-	-	-	-	-	-	-	-	-	-	-	-	-	-

Table S4: Oligonucleotides used in this study

Name	Sequence	purpose
	**mutant genotyping**	
	fgt3	
1412/fgt3_F	ATGTCGAGCTGTGCCTCTGCTCAGC	dCAPS of *fgt3*, PvuII cuts WT
725/fgt3_R	GGAATTCGCTAACGAGGCTTTCC	
	hsfa3-1	
2626/hsfa3genoF	ACTCATTCTTCTTCTTCTTCTTCTTTCAG	genotyping Salk_011107
2627/hsfa3genoR	AGAAGTTATTATAAGATCCAATCGAGGCC	
1537/ Lbb1.3 SALK	ATTTTGCCGATTTCGGAAC	
	hsfa2	
2628/hsfa2genoI	AAACCACCCCAGTACATTAAAAACGTCC	genotyping Salk_008978
2629/hsfa2geno forward	AATCTTGGAATGATAAGTAAGGACTCTGCC	
2630/hsfa2geno R	GAACGTCATCATCTGCTGCTGTCTC	
	**Cloning of Constructs used in this study**	
	gHSFA3	
2410/HSFA3-Prom-f	AAGGCGCGCCAATACCCCCGGTCCATTCAAAAGTTG	genomic HSFA3 fragment
2418/HSFA3-3prime-r	AGAGCTCTTAATTAATGATTAAACCAGACAGCTTCAGAGAG	
	pHSFA3::3xFLAG-HSFA3	
2410/HSFA3-Prom-f	AAGGCGCGCCAATACCCCCGGTCCATTCAAAAGTTG	pHSFA3 fragment
2420/HSFA3-Prom-r	TACCGGTTTGATATAGTAGAAAATTTACGGGTTTTAGTGAG	
2419/HSFA3-3xFlagFw	AACCGGTATGGACTACAAAGACCATGACGGTGATTATAAAGATCATGATATCGATTACAAGGATGACGATGACAAGGGAGCAGGAGCAATGAGCCCAAAAAAAGATGCTGTTTCTAAACC	3xFLAG-HSFA3 + 3'region
2418/HSFA3-3prime-r	AGAGCTCTTAATTAATGATTAAACCAGACAGCTTCAGAGAG	
	pHSFA2::3xFLAG-HSFA3	
2624/pHSFA2-AscI-F	AGGCGCGCCCTGTTTGGTTATCGGGTGAGAGAAAAATTG	pHSFA2 fragment
2625/pHSFA2-AgeI-R	TACCGGTTTTCGTTGTTTATCTCAAATCCATAAGCTCAG	
	pHSFA3::HSFA2-YFP	
2786/HSFA2fwAgeI	AACCGGTATGGAAGAACTGAAAGTGGAAATGGAGG	HSFA2-YFP + 3'region
2787/HSFA23primeR	AGCGGCCGCCTTATAGTTACGTGTTGTGTTGTGTGC	
	pHSFA3::3xFLAG-HSFA1D	
2810/3xFLAG-HSFA1D	AACCGGTATGGACTACAAAGACCATGACGGTGATTATAAAGATCATGATATCGATTACAAGGATGACGATGACAAGGGAGCAGGAGCAATGGATGTGAGCAAAGTAACCACAA	3xFLAG-HSFA1D + 3'region
2811/HSFA1D-3rev	TGCGGCCGCATTAGTCTCGGTTTGGTTTTCAGGG	
	Yeast Two Hybrid clones	
HSFA2_attB_F	GGGGACAAGTTTGTACAAAAAAGCAGGCTCTATGGAAGAACTGAAAGTGGAAATG	HSFA2 full cDNA Gateway®
HSFA2_attB_R	GGGGACCACTTTGTACAAGAAAGCTGGGTTTAAGGTTCCGAACCAAGAAAAC	
HSFA3_fw_pGBKT7	atggaggccgaattcATGAGCCCAAAAAAAGATGCT	HSFA3 full cDNA in pGBKT7
HSFA3_rev_pGBKT7	caggtcgacggatccCTAAGGATCATTCATTGGCGT	
HSFA3_fw_pGADT7	gaggccagtgaattcATGAGCCCAAAAAAAGATGCT	HSFA3 full cDNA in pGADT7
HSFA3_rev_pGADT7	gagctcgatggatccCTAAGGATCATTCATTGGCGT	
HSFA2_fw_pGBKT7	atggaggccgaattcATGGAAGAACTGAAAGTGGA	HSFA2 truncated cDNA in pGBKT7
HSFA2_trunc-r-pGBKT7	caggtcgacggatccTCCTTCATTCaATCAAACTCT	
HSFA2_fw_pGADT7	gaggccagtgaattcATGGAAGAACTGAAAGTGGA	HSFA2 truncated cDNA in pGADT7
HSFA2-trunc-r-pGADT7	gagctcgatggatccTCAAACTCTTaATCATGTAA	
HSFA3_trunc_attB_F	GGGGACAAGTTTGTACAAAAAAGCAGGCTCTATGAGCCCAAAAAAAGATGCTG	HSFA3 truncated cDNA Gateway®
HSFA3_trunc_attB_R	GGGGACCACTTTGTACAAGAAAGCTGGGTTTATTCATACTTCACCACCTCCCCTC	
	pIX-in vitro expression clones	
4114-pHALOHsfA1a-	GGGGACAAGTTTGTACAAAAAAGCAGGCTTAATGTTTGTAAA	HSFA1a

Name	Sequence	purpose
attB1_F1	TTTCAAATAC	
4115 / pHALO-HsfA1a-attB2_R1	GGGGACCACTTTGTACAAGAAAGCTGGGTTCTAGTGTTCTGTTTCTGATG	HSFA1a
4116 / pHALO-HsfA1b-attB1_F1	GGGGACAAGTTTGTACAAAAAAGCAGGCTTAATGGAATCGGTTCCCGAATC	HSFA1b
4117 / pHALO-HsfA1b-attB2_R1	GGGGACCACTTTGTACAAGAAAGCTGGGTTTTATTTCCTCTGTGCTTCTG	
4118 / pHALO-HsfA1d-attB1_F1	GGGGACAAGTTTGTACAAAAAAGCAGGCTTAatggATGTGAGCAAAGTAAC	HSFA1d
4119 / pHALO-HsfA1d-attB2_R1	GGGGACCACTTTGTACAAGAAAGCTGGGTTTTACCTTGAGAGATCTAAG	
4120 / pHALO-HsfA2-attB1_F1	GGGGACAAGTTTGTACAAAAAAGCAGGCTTAATGGAAGAACTGAAAGTG	HSFA2
4121 / pHALO-HsfA2-attB2_R1	GGGGACCACTTTGTACAAGAAAGCTGGGTTTTAAGGTTCCGAACCAAG	
4122 / pHALO-HsfA3-attB1_F1	GGGGACAAGTTTGTACAAAAAAGCAGGCTTAATGAGCCCAAAAAAAGATG	HSFA3
4123 / pHALO-HsfA3-attB2_R1	GGGGACCACTTTGTACAAGAAAGCTGGGTTCTAAGGATCATTCATTGG	
4124 / pHALO-HsfA7a-attB1_F1	GGGGACAAGTTTGTACAAAAAAGCAGGCTTAATGATGAACCCGTTTCTC	HSFA7a
4125 / pHALO-HsfA7a-attB2_R1	GGGGACCACTTTGTACAAGAAAGCTGGGTTTTAGGAGGTGGAAGCCAAAC	
**qPCR (Gene expression)**		
1547/F-AT4G26410	GAGCTGAAGTGGCTTCAATGAC	AT4G26410 Reference gene
1548/R-AT4G26410	GGTCCGACATACCCATGATCC	
352/LP-Hsa32	CTGATGCGAAGTTGGTTGAG	HSA32
353/RP_Hsa32	GCACATAACATCAGACACATACGA	
637/HSP22 REV	TTCAGGAGATAGTTTCGTGAGGTTA	HSP22
638/HSP22 FOR	ATTCTGGAGACAGTTCAAGCTACCT	
359/HSP18.2_qPCR_F	ACAAACGCAAGAGTGGATTGGA	HSP18.2
360/HSP18.2_qPCR_R	GCTCCTCTCTCCGCTAATCTGC	
363/HSP21_qPCR_F	TGGACGTCTCTCCTTTCGGATTGT	HSP21
364/HSP21_qPCR_R	TGCACGAATCTCTGACACTCCACT	
267/HSP101F	ATGACCCGGTGTATGGTGCTAG	HSP101
268/HSP101R	CGCCTGCATCTATGTAAACAGTG	
265/HSP70F	CCGTCTTCGATGCTAAGCGTCT	HSP70
266/HSP70R	AACCACAATCATAGGCTTCTCACC	
633/APX2 REV	ACTCCTTGTCAGCAAACCCGAG	APX2
634/APX2 FOR	CTTGATGATCCTCTCTTTCTCCCA	
2920/Mips2_qRTPCR-r	AGCCAATAGATTCTCCGTCGTATC	MIPS2
2921/Mips2_qRTPCR-f	AAGGACATGAGGGGAGTTTAAGG	
2947/HSFa1E_R	TTCGCCGTCGCTACAGATTC	HSFA1E
2948/HSFa1E_F	CGAATATAAATCTCTGTAAGGTTTGCG	
4150/LACS9-F	CACGAAAGAGCAAGCCGTGAAAG	LACS9
4151/LACS9-R	ATCGTGATTGTTTAGCCGCCTTC	
4156/LPAT5-F	AGGGCACAGATTACACAGAGGCTA	LPAT5
4157/LPAT5-R	AAGTGAGCAACTCAGTTCTTGCAAGC	
4158/TPR1-F	ACGACGGATCAAACAAGGAGAAAGC	TPR1
4159/TPR1-R	TATGGATCGAAGCTCTATAGACTCAGG	
4160/Myb86-F	TCTGTCCCTAAACTCGCAGGTTTGCA	MYB86
4161/Myb86-R	AATGATCAAGCTTTCTTCGTCTTGAGAG	
4154/DGS1-F	GAGCAACCGACGAGTGGGATTTAG	DGS1
4155/DGS1-R	TTTGCTGCTGTGGCCTTCCTAAC	
315/Hsa32uspl_F	TATGCTTACTGTGAGAATGCCTTTGT	HSA32 unspliced
653/HSA32-3UTR	Ttcttacagcatcaaagaagca	
2302/Hsp21_unspliced_transcript_F	TTAATCTAACCACAGGATTGTTGGATC	HSP21 unspliced

Name	Sequence	purpose
364/HSP21_qPCR_R	TGCACGAATCTCTGACACTCCACT	
634/APX2 FOR	CTTGATGATCCTCTCTTTCTCCCA	APX2 unspliced
2210/APX2_unspl_R	AGAAGGCATCCTCATCCTGAGAG	
2725/HSP101_unsp_F	CTGACTCTTGTGGTTGCTTTCT	HSP101 unspliced
268/HSP101R	CGCCTGCATCTATGTAAACAGTG	
**qPCR (transcription factor-ChIP)**		
1259/HSP22-3kb_FWD	CGTTGGACTTGGCCTTAGAT	HSP22-1
1260/HSP22-3kb_REV	TGACTGCTCCCTGATTCTTG	
688/HSP22-200bp-f	GACTCATCACAAACAGAATTGGTC	HSP22-2
689/HSP22-200bp-r	TGTTGTGCCTTTAAGGGTCTTC	
1921/HSP22_TSS-60-f	CACAACAAACTTATCCAACG	HSP22-3
1922/HSP22_TSS-60-r	GGTAGAGTTTTGCAGAGAGA	
2225/Mu1Cf	TTCTTCTCCATCGACACTCTCTC	Mu1C
2062/MU1Crev	ATGATGATCCAACGACCAACCAC	
2518/HSP101 -3,2kb-f	CTCTCAAAAAGTGTACCTCCA	HSP101-1
2519/HSP101 -3,2kb-r	GAGCTCCAAGAAAAGGCCAT	
2669/HSP101 -348bp-f	CGTAAATCTTTGGATTTTGA	HSP101-2
2670/HSP101 -348bp-r	TTTCTCCTCTTTTGCCGCAT	
2671/HSP101 -136bp-f	ACATCTACCTGTCGGATCAA	HSP101-3
2672/HSP101 -136bp-r	TCTGGAAAGATAGAGAACTA	
1255/HSP18.2 -3kb_f	GCCCCTAGGGATTTCGACTA	HSP18.2-1
1256/HSP18.2 -3kb_r	CCCGTAAATAAAGCGGTCAA	
1938/HSP18.2 -262bp-f	AGGATAATACAACAACAAAG	HSP18.2-2
1939/HSP18.2 -262bp-r	CACTGTGGTGAAATGACCAG	
1940/HSP18.2 -125bp-f	TATGTGTTCTAAAGACTCCA	HSP18.2-3
1941/HSP18.2 -125bp-r	GTTAGAGGATGAAGAGAGAA	
2324/HSA32 -5kb_F	TGGGTGTGTCCATTTACGTC	HSA32-1
2325/HSA32 -5kb_R	TGTAGGTATCGATGTTGGAG	
694/HSA32 -200bp_f	CATCAACGAGACGGGATTTAG	HSA32-2
695/HSA32 -200bp_r	GATTCTTGACAGAGCAGCCAAG	
1907/HSA32 -75bp_F	TATCCTAAATCCCGTCTCGTT	HSA32-3
1908/HSA32 -75bp_R	ATGTCCAAGTCGAAGAATGG	
1857/APX2 -3.2kb_F	GGATATCAAACCCAACTTGAAGAGAG	APX2-1
1858/APX2 -3.2kb_R	ATAATCTGAGCAAAAGATAAAACACGG	
1239/APX2 HRE1-F	ACGTGGTGTGTATCTGTTGGA	APX2-2
1240/APX2 HRE1-R	AGTCTTCTTTTGGAGATGGACGGT	
1854/APX2 +40bp_F	TCGATAGGTTCTCCATTCTCTTTAGG	APX2-3
1855/APX2 +40bp_R	TTCCTCTTGCATCTCTGAACAGC	
**qPCR (H3K4me3-ChIP)**		
1259/HSP22-3kb_FWD	CGTTGGACTTGGCCTTAGAT	HSP22-1
1260/HSP22-3kb_REV	TGACTGCTCCCTGATTCTTG	
675/4g10250R_ATG_chip/hsp22	AGTCCTAATGGGATTCTCTCCA	HSP22-2
676/4g10250F_ATG+50_chip/hsp22	GTTGCTTGGAAACATCAAAACAAG	
2518/HSP101 -3,2kb_F	CTCTCAAAAAGTGTACCTCCA	HSP101-1
2519/HSP101 -3,2kb_R	GAGCTCCAAGAAAAGGCCAT	
2675/HSP101 +112bp_F	TCTGCTTGATTCTCTGCAA	HSP101-2
2676/HSP101 +112bp_R	ACACACAAATGAGAACAAGA	
1857/APX2 -3.2kb_F	GGATATCAAACCCAACTTGAAGAGAG	APX2-1
1858/APX2 -3.2kb_R	ATAATCTGAGCAAAAGATAAAACACGG	
1869/APX2 +510bp_F	CTGTTCCCTATTCTGTCATATGCTG	APX2-2
1870/APX2 +510bp_R	ACCCTTGATTCTATGGTTCTACCTC	
698/HSA32 +514bp_f	TGCTTCGTAGTGGTCCTTCAG	HSA32-1

Name	Sequence	purpose
699/HSA32 +514bp_r	CCCAACTGCTTACACTCCTGC	
800/HSA32 +838bp_f	AACACCGTTCAGCCTTTCTG	HSA32-2
801/HSA32 +838bp_r	CTCGGTCAAGCGGTAAGAAG	
