# Supplementary material for: Heteromeric HSFA2/HSFA3 complexes drive transcriptional memory after heat stress in Arabidopsis
Source: Nat Commun. 2021 Jun 8;12:3426. doi: 10.1038/s41467-021-23786-6 (PMC8187452; doi:10.1038/s41467-021-23786-6)
Supplement: Supplementary file 2 — Descriptions of Additional Supplementary Files [file 41467_2021_23786_MOESM2_ESM.pdf]

Descriptions of Additional Supplementary Files

Supplementary data 1
Description: Log2 fold change relative to no-HS expression of 1-0-0 up (Col) and 1-1-1 up (Col) genes in Col, hsfa2, hsfa3-1, hsfa2 hsfa3-1.
